# The interplay between androgens and the immune response in polycystic ovary syndrome

**DOI:** 10.1186/s12967-023-04116-4

**Published:** 2023-04-16

**Authors:** Sania Shabbir, Emaan Khurram, Vedhika Sathya Moorthi, Youssef Tamer Hassan Eissa, Mohammad Azhar Kamal, Alexandra E. Butler

**Affiliations:** 1 Royal College of Surgeons in Ireland Bahrain, Adliya, 15503 Bahrain; 2 Department of Pharmaceutics, Prince Sattam Bin Abdulaziz University, Al-Kharj, 11942 Saudi Arabia

**Keywords:** Polycystic ovary syndrome, Androgens, Metabolic pathways, Inflammation, Cytokines

## Abstract

Polycystic ovary syndrome (PCOS) is a metabolic-reproductive-endocrine disorder that, while having a genetic component, is known to have a complex multifactorial etiology. As PCOS is a diagnosis of exclusion, standardized criteria have been developed for its diagnosis. The general consensus is that hyperandrogenism is the primary feature of PCOS and is associated with an array of physiological dysfunctions; excess androgens, for example, have been correlated with cytokine hypersecretion, adipocyte proliferation, and signaling pathway dysregulation. Another key feature of PCOS is insulin resistance, resulting in aberrant glucose and fatty acid metabolism. Additionally, the immune system plays a key role in PCOS. Hyperandrogenism stimulates some immune cells while it inhibits others, thereby disrupting the normal balance of immune cells and creating a state of chronic inflammation. This low-grade inflammation could contribute to infertility since it induces ovarian dysfunction. This dysregulated immune response in PCOS exhibits autoimmunity characteristics that require further investigation. This review paper examines the relationship between androgens and the immune response and how their malfunction contributes to PCOS.

## Introduction

In premenopausal women, polycystic ovary syndrome (PCOS) is one of the most common endocrine-metabolic disorders. A patient with PCOS commonly has hyperandrogenism and dysfunction of the ovaries. Abdominal adiposity, obesity, and insulin resistance are commonly associated with this condition [1]. PCOS women tend to have prolonged and/or irregular menstrual cycles leading to anovulatory infertility [2]. They are often infertile due to anovulation; if they manage to become pregnant, they are more likely to suffer pregnancy complications, such as miscarriage or premature delivery. While the cause of PCOS is unknown, it is associated with cardiovascular disease and type-2 diabetes mellitus (T2DM), which lead to elevated androgens and insulin resistance [2]. Furthermore, PCOS patients can develop a host of complications, including metabolic syndrome, depression, anxiety, sleep apnea, and endometrial cancer [2].

The National Institutes of Health (NIH), the Androgen Society, and the Rotterdam criteria have been used in diagnosing PCOS; all three have hyperandrogenism as a central facet of their diagnostic criteria [3]. The Rotterdam criteria are the most commonly used and most widely accepted. The Rotterdam criteria state that two out of the following three criteria must be met to diagnose PCOS: hyperandrogenism, polycystic ovaries found on ultrasound, and oligo/anovulation. Clinical and/or biochemical hyperandrogenism can be used for diagnosis; biochemical hyperandrogenism is indicated by an increased free androgen index (FAI) or raised testosterone levels. Patients with the more metabolic phenotype of PCOS are more likely to have both hyperandrogenism and oligo/anovulation. As PCOS is a diagnosis of exclusion, other etiologies, such as thyroid dysfunction, androgen-secreting tumors, and hyperprolactinemia, must be excluded before the diagnosis can be made [4].

Numerous studies have aimed to attain a deeper mechanistic understanding of PCOS, such as its relationship with the immune system and how it affects bodily functions. Several key factors are known to underlie the pathophysiology of PCOS. Firstly, there is dysregulation of metabolic pathways, such as those integral to glucose and fatty acid metabolism. This, in turn, leads to complications such as obesity and T2DM [5]. Obesity is associated with insulin resistance and hyperinsulinemia, while T2DM, by definition, is associated with hyperglycemia [6]. The involvement of androgens with the immune system is crucial to understand as androgen levels are elevated in PCOS, and activation of the immune system stimulates the production of androgens [7]. Furthermore, hyperandrogenism in PCOS seems to be immunosuppressive because it inhibits several immune cells, such as B and T cells [8]. As for the other immune system cells, studies have suggested the association of androgens with neutrophils and dendritic cells [8]. Cytokines, such as tumor necrosis factor-alpha (TNF-ɑ), are influenced by androgens [9, 10]. In addition, several interleukins seem to also be affected by androgens, including interleukin-22 (IL-22), interleukin-1α (IL-1α), and interleukin-6 (IL-6). For example, a study concluded that increased amounts of IL-1ɑ inhibit oestradiol secretion [11]. Additionally, PCOS is closely associated with several other pathways in the body, such as the transforming growth factor β (TGFβ) pathway [12], AKT/PI3 kinase pathway [13], the MAPK cascade [14], and the JNK/ERK pathway [15]. Through several studies, these pathways have been suggested to be connected to hyperandrogenism in PCOS. In PCOS patients, adipocytes produce higher than average amounts of androgens. Not only are androgens damaging to adipocytes, but they also stimulate the secretion of steroidogenic cells [16]. Studies have suggested that abnormalities in steroidogenic cells contribute to the hypersecretion of androgens in PCOS [17]. Additionally, the hyperandrogenism of PCOS leads to more significant amounts of abdominal adipose tissue both in obese and normal-weight PCOS women [14]. Furthermore, there is increased oxidative stress (OS) present in PCOS women, contributing to obesity and insulin resistance [18].

This review discusses the association of androgens with metabolic pathways, immune cells, and their role in PCOS. We have comprehensively summarized these relationships, incorporating the most up-to-date literature, and have provided further ideas for exploring the connection between the immune system and PCOS.

## Dysregulation of metabolic pathways in PCOS

**Fig. 1:**
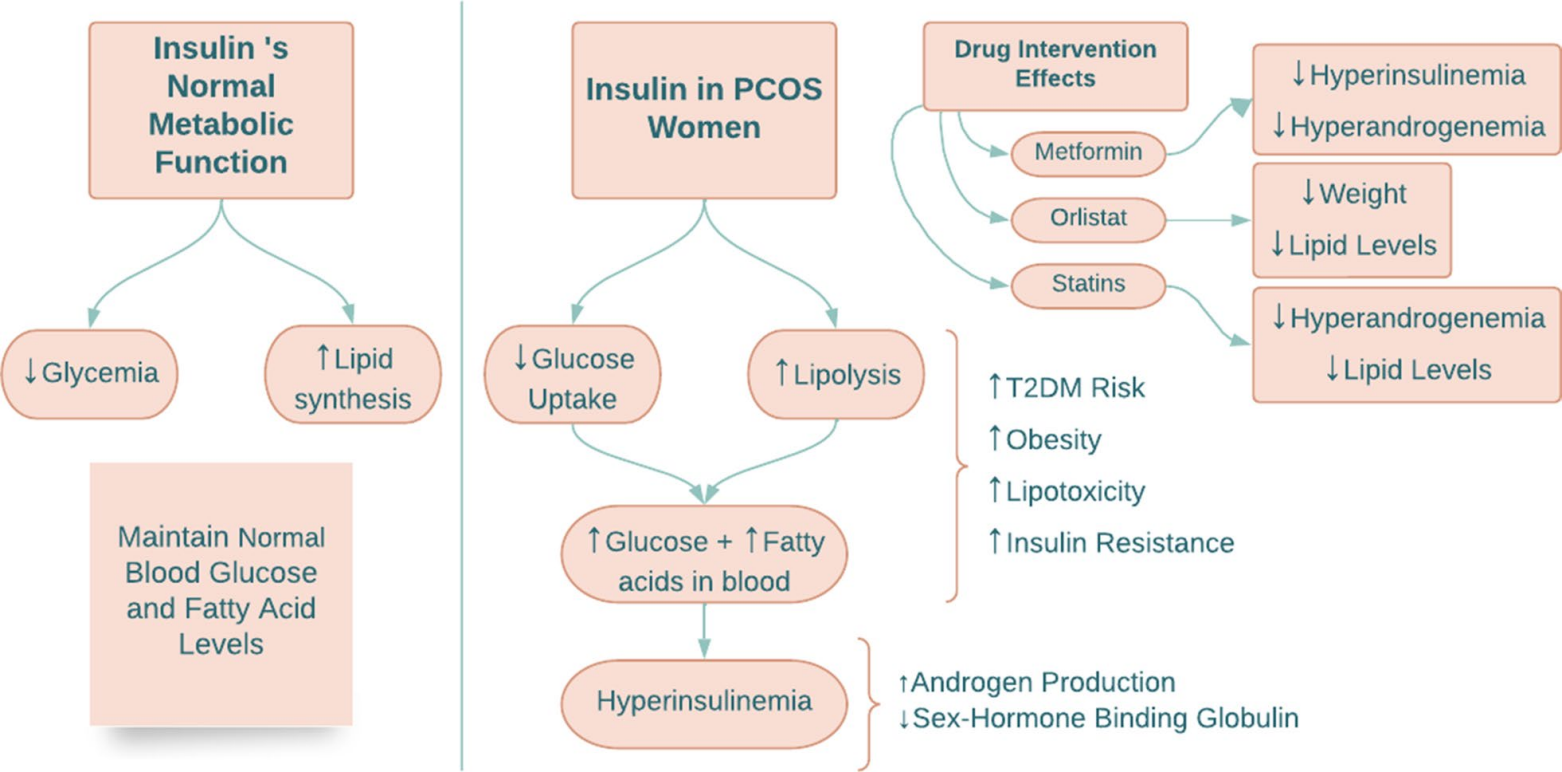
A schematic summarizing insulin action in PCOS. *T2DM* type two diabetes mellitus

Glucose and fatty acid metabolic pathways are connected via the action of insulin. Insulin’s effect on glucose metabolism is via inhibition of gluconeogenesis and glycogenolysis, resulting in a decrease in circulating glucose levels [6]. Insulin also increases lipid synthesis and inhibits lipolysis by suppressing hormone-sensitive lipase [6]. However, in PCOS women, early steps in the insulin pathway, such as the high uptake of glucose and inhibition of lipolysis, are decreased, resulting in increased glucose and fatty acid levels in the circulation (Fig. 1) [6]. Hyperinsulinemia and insulin resistance are standard characteristics of women with PCOS; these could either be primarily due to genetics or secondary to obesity [6]. The risk of T2DM through obesity may increase due to insulin resistance and the metabolic phenotype [6]. Free fatty acids accumulate in tissues due to increased lipolysis causing insulin resistance and lipotoxicity [6]. In a study by Whigham et al., a breath analysis indicated decreased lipid oxidation in PCOS women compared to non-PCOS women, suggesting that PCOS women have difficulty switching from glucose oxidation to lipid oxidation [5]. This emphasizes that these women have lipid stores that are not being utilized, contributing to obesity and T2DM [5].

Insulin and androgen levels are positively correlated: high insulin levels result in increased androgen levels. Insulin acts as a co-gonadotropin by increasing ovarian theca cells’ production of androgens. It also increases the sensitivity of the adrenal cortex to adrenocorticotropic hormone (ACTH), further boosting androgen levels [10]. In a study by Baptiste et al. in obese PCOS women, reducing the amount of insulin with diazoxide resulted in increased levels of sex-hormone binding globulin (SHBG) [6]. This implies that insulin inhibits the secretion of SHBG from the liver, resulting in high levels of free androgens, and explains why obese PCOS women with hyperinsulinemia have low SHBG levels. These results are not only seen in obese PCOS women but also in PCOS women who are lean and normoinsulinemic. Following the administration of diazoxide in PCOS women, their high levels of free androgens were decreased due to insulin suppression. However, lowering insulin levels by diazoxide in non-obese women without PCOS did not influence androgen levels [6]. This implies that, regardless of whether a woman with PCOS is obese/lean or has high/normal insulin levels, insulin action directly affects androgen levels.

In a study by Kuang et al., how the insulin signaling pathway in PCOS women is affected by serum inflammatory cytokines and berberine was investigated. Cytokines were studied in cells (treated with berberine, an insulin-sensitizing agent) from 78 infertile women who underwent in vitro fertilization (IVF) [19]. IL-1ɑ, IL-6, and IL-17ɑ were all elevated in the PCOS group causing the PCOS women to be in a prolonged subclinical inflammatory state [19]. Due to these elevated interleukins, regular ovulation and fertilization, together with glycolipid metabolism, are impaired, and insulin resistance is promoted, all of which contribute to the metabolic perturbations seen in PCOS. Berberine improves the sensitivity of insulin and increases glucose uptake in PCOS women by modulating insulin receptor substrate-1, thereby exerting a modulatory effect on PCOS [19].

Metformin and rosiglitazone, two insulin-sensitizing drugs, both positively impact the metabolic and hyperandrogenic state in PCOS women [6]. In a study by Baptiste et al., both drugs were used in non-obese women with PCOS, demonstrating that even in PCOS women who are not obese and have normal levels of insulin, hyperandrogenemia is connected with insulin resistance [6]. Metformin reduced insulin levels below normal by decreasing liver glucose production and reducing high androgen levels, thereby decreasing the incidence of T2DM in PCOS patients [20]. It is prescribed with a starting dose of 500-850 mg per day that can be increased to 2000 mg as needed [20]. Rosiglitazone, a PPARγ agonist, also reduced the androgen levels without changing insulin levels, which suggests that PPARγ agonists may dampen the hyper-responsiveness of androgens to insulin [6].

Weight loss medications such as orlistat were used in a randomized controlled trial to compare their efficacy with metformin and exercise [21]. In the study, ninety obese PCOS women were randomly assigned to three treatment groups. Two groups received either orlistat or metformin plus lifestyle intervention, and one group was assigned lifestyle intervention only [21]. Orlistat and metformin reduced weight, waist circumference, and body mass index; however, orlistat had fewer side effects and improved lipid profiles [21]. Therefore, both drugs positively affected weight loss and ovulation, but orlistat had fewer side effects and was better tolerated than metformin [21].

Statins, widely prescribed cholesterol-reducing drugs, were used in a three-month study in PCOS women, achieving a significant decrease in hyperandrogenemia and lipid levels [22]. In another study of statins, where twelve different trials were compared, the results showed an overall reduction in testosterone and other androgen hormones, as well as in low-density lipoproteins [23]. In addition, statins normalize the follicle-stimulating hormone (FSH) and luteinizing hormone (LH) ratio, which promotes estrogen production over testosterone, hence allowing follicle maturation and release [23]. However, there are concerns about the use of statins in premenopausal women as they are known to be teratogenic [22] and have conflicting effects on glucose [23]; some studies show a reduction in insulin levels after statin administration while others show an association with hyperinsulinemia [23]. More research is required to determine the optimal therapeutic strategy for the use of statins in PCOS [23].

## Relationship of hyperandrogenism to the immune response in PCOS

A key aspect of PCOS is the immune system's involvement in its pathophysiology. Hyperandrogenism affects the immune cell subtypes in different ways, but generally, excess androgen levels in the body stimulate certain types of immune cells, such as neutrophils, whilst others like dendritic cells, are inhibited by hyperandrogenism. These immune cells, in turn, produce a wide range of cytokines that contribute to many of the features of PCOS [8]. This section will additionally examine the role of the complement system in PCOS development.

The nature of PCOS ultimately depends on the interplay between immune system activity and androgen levels. Gleicher et al. chose to avoid measuring the ovarian reserve (OR) of the female subjects, which they defined as the “sum of all remaining follicles,” [7] as it included a large number of follicles that do not actually grow. Instead, they chose to study the functional ovarian reserve (FOR), which refers to “the pool of small growing follicles” [7]. In this study, subjects were divided into three groups: females with an age-dependent decrease in FOR, females with a premature decline in FOR, and controls with normal FOR, and levels of immune activation and androgens were assessed [7]. The key finding was that the controls with normal FOR, who primarily also showed increased immune system activation, had heightened amounts of testosterone compared to females with decreased FOR [7]. The lower testosterone levels in females with decreased FOR may also be due to lower overall numbers of theca cells, indicative of reduced androgen production. The authors explained these findings by suggesting that females with normal FOR had increased androgen levels because the magnitude of their immune system’s activity was positively correlated with the production of an androgen-producing factor (APF) [7]. As a result, in females with decreased FOR, the decreased immune function is associated with diminished levels of APF, which in turn is correlated with decreased androgen levels [7]. Based on the suggestion that immune system function stimulates androgen synthesis, androgens would need to be immunosuppressive to function as part of a negative feedback process, thereby regulating androgen levels [7]. Using this model of immune activation-stimulated androgen production to explain PCOS, the authors suggested that autoantibodies induced by inflammation in PCOS lead to excessive immune system activation which, in turn, results in increased synthesis of APF and consequent hyperandrogenism [7]. According to Hu et al., the relationship between the inflammatory state and the production of autoantibodies in PCOS can be explained by considering that the inflammation is associated with increased levels of pro-inflammatory immune cells and inhibition of anti-inflammatory cells; this “immune microenvironment imbalance” then drives the production of autoantibodies [24]. Certain studies have also investigated the effect of androgens on specific types of immune cells, which are categorized into myeloid, lymphoid, and dendritic cells.

### Macrophages in the pathogenesis of PCOS

In the ovaries and adipose tissue, macrophages are the most abundant immune cell and are necessary for balancing destructive and protective cell-mediated immunity in inflammation [24]. Macrophage levels vary throughout the menstrual cycle and are highest during ovulation and the luteal phase; this suggests that macrophages are under the hormonal regulation of progesterone [24]. Women with PCOS commonly present with obesity and insulin resistance, pathophysiological states characterized by macrophages becoming a pro-inflammatory M1 phenotype from an anti-inflammatory M2 phenotype [24]. M1 macrophages produce inflammatory cytokines, such as IL-1, IL-6, and TNF-ɑ, that are present in high levels in both the serum and follicular fluid of PCOS women [24, 25]. The hyperandrogenism found in PCOS likely results in the conversion of macrophages to the M1 state, increasing cytokine levels, thereby amplifying PCOS symptoms such as insulin resistance, androgen production, and the imbalance in the hypothalamic-pituitary-ovarian axis secretion [24, 25]. The effects of these macrophage-produced cytokines, as well as the cytokines produced by other immune cells, are discussed in more detail in the next section in order to highlight how the immune system is dysfunctional in PCOS.

### Neutrophils and their effects in PCOS

**Fig. 2:**
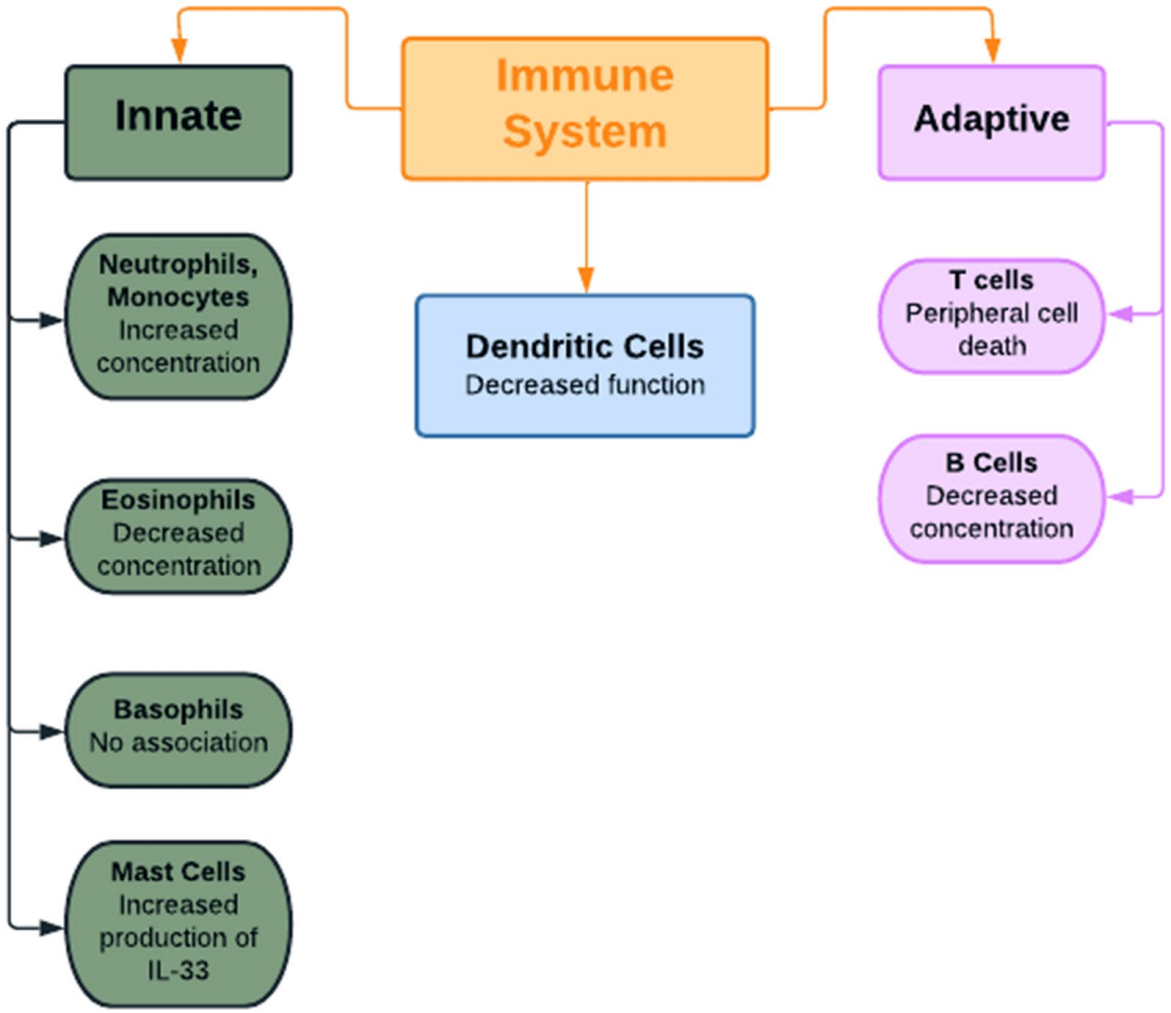
A summary of the effects of hyperandrogenism on different cell types of the innate and adaptive immune systems. *IL-33* interleukin-33

Androgens are associated with an increased rate of production of neutrophils from myeloid precursor cells; therefore, an excess of neutrophils (neutrophilia) is often seen in patients with PCOS (Fig. 2). This concept is supported by a study showing the reduction in neutrophil levels following administration of 62.5 mg of flutamide, an anti-androgen drug, for 3 months [8, 26]. The excess neutrophils, in turn, produce a wide range of cytokines, such as TNF-ɑ, TGFβ, IL-6, IL-1α, and IL-1β, that contribute to the pathophysiology of PCOS [27]. Additionally, neutrophils ingest and destroy defective and dead cells present in excess fat, which eventually results in chronic low-grade inflammation [24]. Usually, PCOS is treated with the oral contraceptive pill, which increases neutrophil levels even further, though metformin is known to treat immune disorders and can be utilized to decrease neutrophil levels [24].

### Other myeloid cells associated with PCOS

While androgens have been associated with increased levels of neutrophils and monocytes, they have been correlated with a decrease in the concentration of eosinophils; this relationship was reported after observing a rise in eosinophils following gonadectomy in mice [8]. Conversely, testosterone does not appear to affect the numbers of mast cells but does promote mast cell production of IL-33, which, in turn, drives the production of basophils as well as innate lymphoid cells [8]. No association between basophils and androgens has been reported [8]. Overall, it seems that androgens stimulate the rapid non-specific immune response to pathogens and, as a consequence, hyperandrogenism in PCOS may be associated with increased phagocytosis and other functions carried out by the innate immune system.

### The role of the complement system in PCOS

Another important component of innate immunity is the complement system, which regulates inflammation via a stepwise series of proteins known as the complement cascade [28]. The complement cascade has three activation pathways: the classical, alternate, and lectin pathways. C3, a central component, is increased in patients with metabolic diseases such as T2DM [28]. The terminal pathway contains the C5 component, which forms the terminal complement complex (TCC). In a case–control study by Lewis et al. comparing fasting and postprandial states in PCOS and control women to establish if the complement system was activated in PCOS patients, plasma complement proteins involved in both the activation and terminal complement pathways were determined [28]. C3, C3a(desArg), the C3a(desArg)/C3 ratio, and the TCC levels were increased in PCOS versus non-PCOS women and were particularly high in PCOS patients with obesity and insulin resistance [28]. Interestingly, C3 and C3a(desArg) levels decreased in response to metformin and statin treatment.

### The relationship between androgens and lymphoid cells

A large body of research has investigated the association between androgen levels and the activity of B and T cells. Many studies similarly concluded that androgens negatively correlate with B cell levels [8]. A gonadectomy in mice, with a subsequent fall in androgen levels, stimulates B cell production, whereas administration of androgens (testosterone or 5ɑ-dihydrotestosterone (DHT)) inhibits B cell synthesis [8]. Similar results were also found in human subjects; a high testosterone level in males was associated with low B cell function and inadequate antibody production [8]. It was suggested that high androgen levels promote TGFβ production, which inhibits interleukin-7 (IL-7) and therefore opposes B cell production [8]. In women with PCOS, it would therefore be expected that hyperandrogenism would suppress B cell activity. As B cells produce IgM, hyperandrogenism would inhibit IgM synthesis, resulting in increased susceptibility to infection [29].

Regarding the effect of androgen on T cell function, two key points are notable. Testosterone deficiency is associated with growth and enlargement of the thymus, whereas an excess of DHT promotes peripheral T cell death [8]. T cells play a role in inducing granular cell growth and selecting ovarian follicles by secreting chemokines and growth factors; they also promote granulosa cell apoptosis by releasing cytotoxic agents [24]. In a study by Wu et al., CD45RO + (memory T cells) were shown to negatively correlate with testosterone levels in theca cells of PCOS women and controls [30]. The PCOS group had very high androgen levels resulting in very low CD45RO + levels, which promotes PCOS development due to impaired natural selection of follicles [24].

Even though androgens generally promote the function of the innate immune system, they seem to have the opposite effect on the adaptive immune system [8] as androgens inhibit the specific antibody-driven response to pathogens resulting in increased susceptibility to infection in PCOS [8]. Additionally, it can be inferred that patients with PCOS may also have a poor response to an infection occurring a second time due to poor function of B cells and impaired conversion into memory cells [8].

### The role of T-cells and biomarkers in inflammation

T helper (Th) cells direct pro-inflammatory and anti-inflammatory immune signals [24]. IL-12 drives the conversion of T cells into Th1 cells, while IL-13 and IL-14 convert T cells to Th2 cells [24]. Androgens and estradiol correlate with inflammation since levels of IL-12 were significantly higher in follicular fluid in PCOS women, while IL-13 levels were decreased according to a study by Gallinelli et al. [32]; this would result in a shift to Th1 cells. Furthermore, due to the many follicles without ovulation in PCOS women, there are increased estrogen levels, which amplify inflammatory cytokine secretion from Th1 cells like IL-6, TNF-α, and interferon-y (IFN-γ) [24]. IL-6 promotes the conversion of Th0 cells to Th17 cells, which are pro-inflammatory in both the blood and kidneys [24]. An increased divergence between the numbers of Th17 and Th2 cells, leaning significantly towards Th17 cells, is common in PCOS women [24]. Hence, there are increased levels of Th1 and Th17 cells in PCOS patients, which leads to an excessive immune response; this suggests an autoimmune origin underlying the development of PCOS. Regulatory T cells (Tregs) play an important role in inhibiting autoimmune diseases, so their malfunction would result in an increased risk of an autoimmune disease. In PCOS patients, the number of Tregs is decreased compared to controls in peripheral blood, so the ratio of Th17/Tregs would increase towards Th17, which results in a chronic inflammatory state [24].

Inflammation is one of the most likely underlying causes of PCOS though this is generally overlooked and is a common thread linking insulin resistance, T2DM, and obesity [10]. To maintain normal ovarian function, it is essential for inflammatory marker levels to be in balance; differing levels of pro-inflammatory and anti-inflammatory cytokines contribute to ovarian dysfunction and improper follicular maturation [10]. Abnormal levels of cytokines, such as TNF-ɑ, IL-6, IL-8, IL-10, IL-18, IL-33, and C-reactive protein (CRP), result in ovarian dysfunction [10]. CRP is the strongest biomarker of inflammation in PCOS women; a high CRP level indicates a potential risk for T2DM development due to high inflammation [10]. CRP is also an important marker for cardiovascular risk and can be used to predict the chance of cardiovascular disease in PCOS patients [10]. Treatments for PCOS usually focus on treating metabolic and ovulatory issues; however, treating the chronic inflammation in PCOS women would help to modulate the associated metabolic and reproductive risk factors [10].

### The effect of hyperandrogenism on natural killer cells

Natural killer (NK) cells are innate immune cells derived from lymphoid progenitors; they kill not only microbes and tumor cells but also regulate other immune cells, such as dendritic cells (DCs) and macrophages. Uterine NK cells are the CD3 − /CD56 + granular lymphocytes, which are unlike any of the NK subtypes in peripheral blood, and have high expression of CD56 [24]. Androgen receptors suppress the expression of IL-12ɑ, which, in turn, reduces the efficiency of NK cells. In a study where the androgen receptor antagonist sorafenib was administered at 30 mg/kg of body weight in mice for 16 days, NK cell function was improved [24, 31]. PCOS women with high androgens have cytokine dysregulation with reduced levels of IL-12ɑ, resulting in fewer NK cells being recruited. This is significant because such a cytokine disorder, with reduced levels of IL-12ɑ, IL-15, IL-18, and CXCL10, impairs fetal-maternal tolerance and maintenance of pregnancy since these cytokines aid in the process of endometrial decidualization [24].

### The inhibition of dendritic cells in PCOS

Dendritic cells (DCs) can be thought of as members of both the adaptive and innate immune systems; they have the phagocytic ability to process antigens and present them to T cells in lymph nodes. They are inflammatory and produce cytokines “such as TNF-ɑ, IL-6, IL-11, IL-12, and IL-23, which, in turn, induce the proliferation of allogeneic T cells and differentiate them to the Th17 and Th1 subtypes” [24]. The number of DCs in follicular fluid in PCOS women is reduced compared to non-PCOS women due to the negative effect that androgens have on DCs [24]. This finding is supported by the fact that gonadectomy in male mice promotes the synthesis of costimulatory substances and major histocompatibility complex (MHC) for dendritic cells, therefore increasing dendritic cell activity [8]. Likewise, males with a hypogonadal condition demonstrated increased dendritic cell activity, further supporting the immunosuppressive influence of androgens on DCs [8]. The result is inadequate levels of DCs to activate Th17 and Th1, leading to failure of ovarian follicle development and maturation [24]. As androgens have an inhibitory effect on DCs, the cytokines produced by DCs are reduced in concentration; however, since androgens also stimulate other immune cells that produce the same cytokines, overall cytokine production is not reduced [24]. Moreover, androgens stimulate increased levels of monocytes but actually inhibit dendritic cells; therefore, it is possible that one of the immune-related actions of androgens is favoring the differentiation of monocytes to macrophages rather than dendritic cells.

To summarize the effect of hyperandrogenism on immune function in PCOS, excess androgen is associated with increased neutrophil activity, greater monocyte function, and high production of IL-33 from mast cells. However, there is a decreased concentration of eosinophils, suppressed B and T cell activity, and inhibition of dendritic cell function [8]. These changes in immune cell levels result in changes in cytokine concentrations which directly contribute to the pathophysiology commonly seen in PCOS.

## The effect of androgens on cytokines

As noted above, hyperandrogenism in PCOS affects immune cells in different ways, but their key function in PCOS is the production of a wide range of cytokines, including TNF-ɑ, IL-1, IL-6, and IL-22. In PCOS, these cytokines have negative effects on many body functions and contribute to many of the clinical features of PCOS, such as insulin resistance, obesity, hypertension, and infertility [9–11].

### The role of tumor necrosis factor-ɑ (TNF-ɑ) in infertility and insulin resistance

**Fig. 3:**
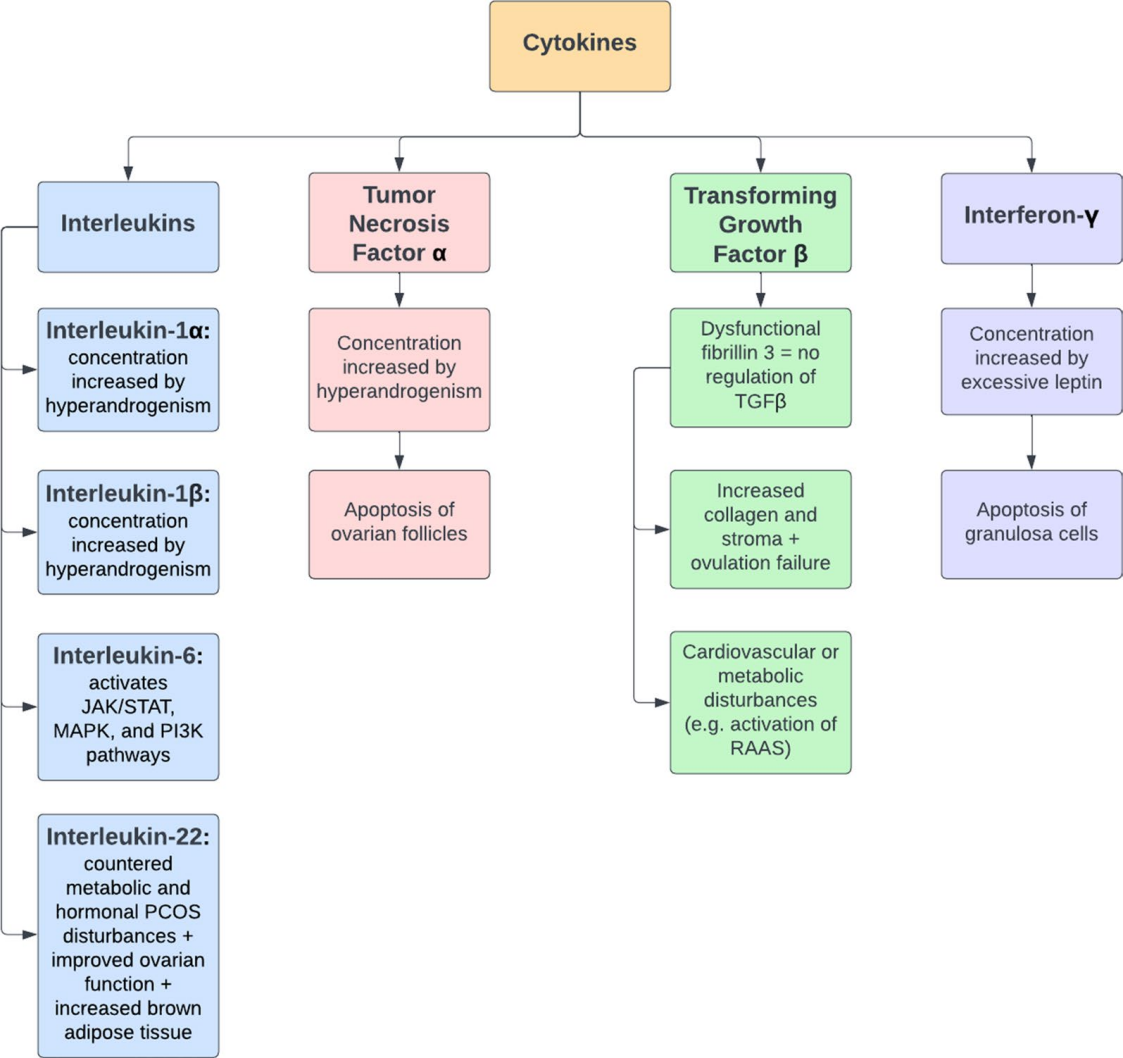
The role of cytokines in the development of PCOS. *RAAS* renin–angiotensin–aldosterone system, *TGFβ* transforming growth factor β

Krishnan et al. investigated the influence of hyperandrogenism on TNF-ɑ synthesis in six female rats. In order to induce high androgen levels, the researchers used an osmotic pump that secreted 83 µg of DHT per day for 90 days [9]. During this period, the levels of TNF-ɑ, IL-1β, FSH, LH, cortisol, and other biomolecules were monitored [9]. The results were compared to six control female rats with normal androgen levels [9]. The most pertinent finding was that the rats with hyperandrogenism had significantly higher levels of TNF-ɑ and IL-1β compared to the control rats (Fig. 3); additionally, the ovaries in the rats receiving excess androgen were smaller and had numerous cysts that resemble PCOS in women [9]. When attempting to explain these results, Krishnan et al. suggested that androgens act as pro-inflammatory agents in PCOS by inducing the nuclear factor of T-cells (NFAT5), which in turn stimulates the expression of TNF-ɑ and IL-1β [9]. The elevated concentration of TNF-ɑ consequently may result in apoptosis or atresia of the ovarian follicles [10]. These findings suggest that the key factor associated with excess TNF-ɑ is the hyperactivation of gene transcription; therefore, it may be beneficial to develop therapeutic agents that repress gene transcription in this case.

**Fig. 4:**
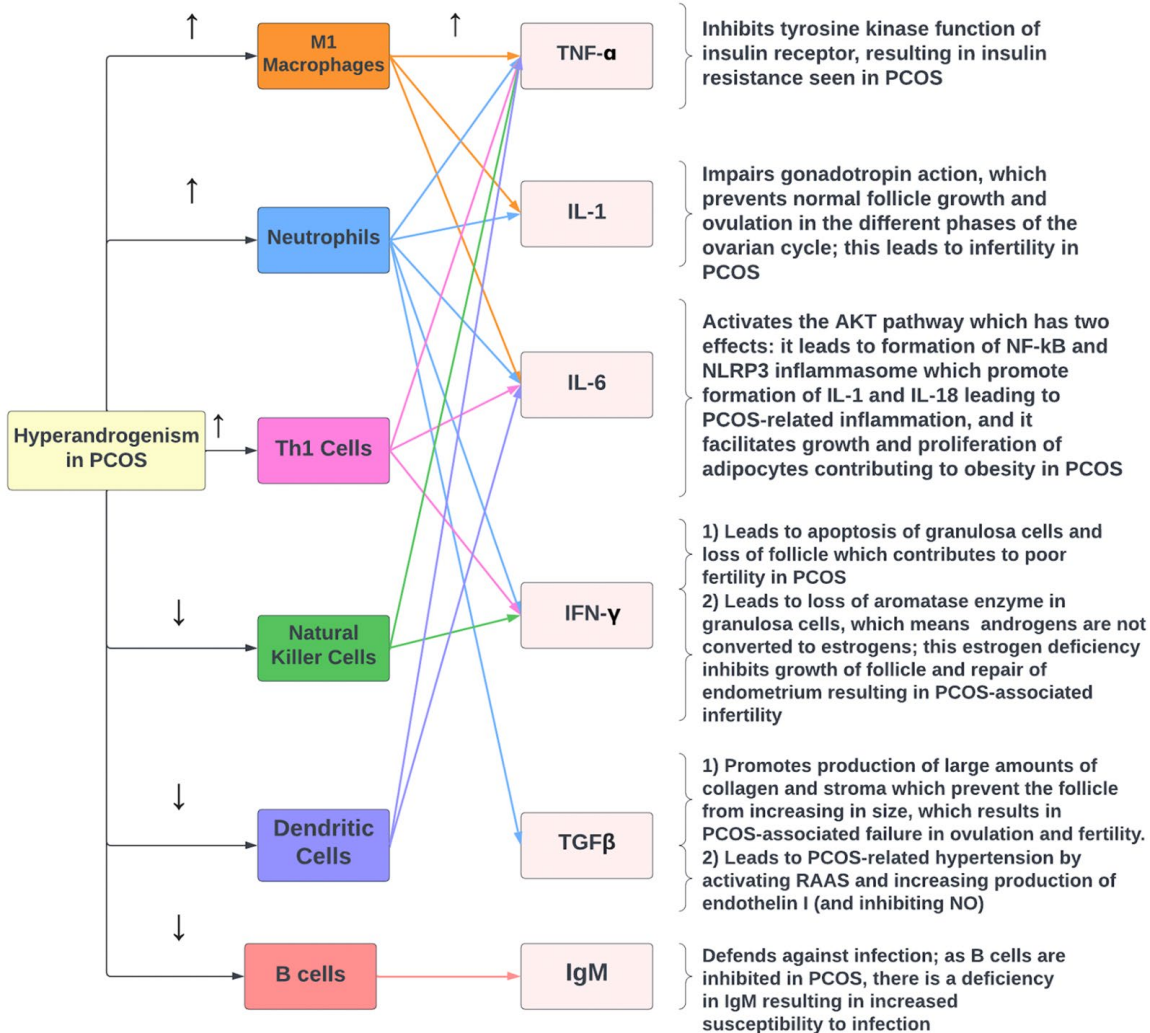
The relationship between hyperandrogenism, the immune system, cytokines, and the features of PCOS. *TNF-α* tumor necrosis factor-α, *IL* interleukin, *Th1 cells* T helper 1 cells, *NF-κβ* nuclear factor-κβ, *IFN-γ* interferon-γ, *TGFβ* transforming growth factor β, *RAAS* renin–angiotensin–aldosterone system, *NO* nitric oxide

As previously mentioned, TNF-ɑ is produced by M1 macrophages, neutrophils, and Th1 cells, which are all induced by the hyperandrogenism in PCOS; as a result, the significant increase in TNF-ɑ then inhibits the tyrosine kinase function of the insulin receptor, impairing the signal transduction pathway for insulin and ultimately leading to the insulin resistance seen in PCOS (Fig. 4) [33]. Additionally, recent research has suggested that high TNF-ɑ levels in PCOS could stimulate nuclear factor kappa B p65 (NF-κBp65) which, in turn, inhibits the endometrial GLUT-4 glucose transporter, possibly contributing to PCOS-associated hyperglycemia [33].

### The relationship between interleukin-1 (IL-1) and infertility in PCOS

**Fig. 5:**
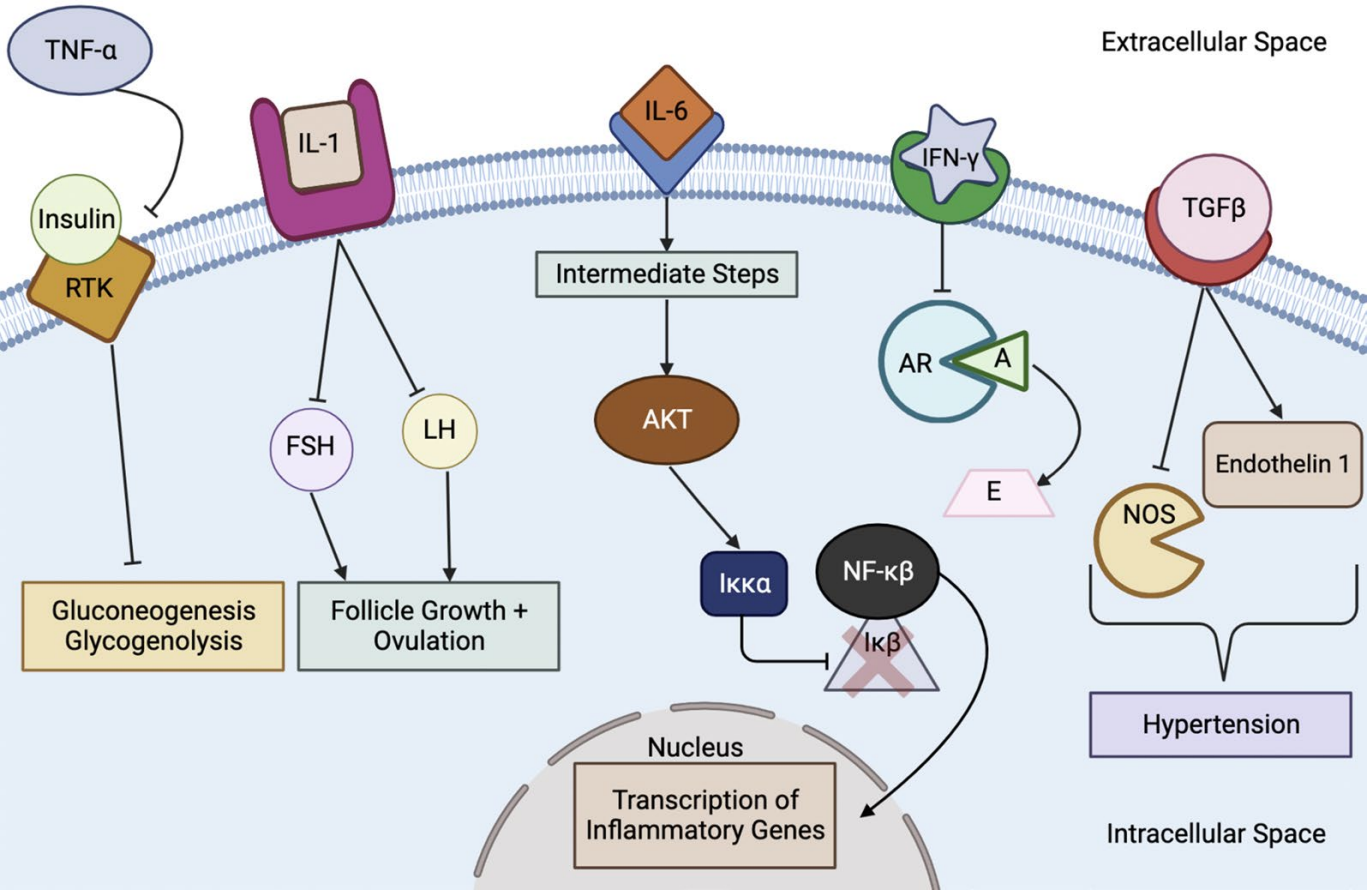
Cytokine Signaling Cascade in PCOS. *TNF-α* tumor necrosis factor-α, *RTK* receptor tyrosine kinase, *IL* interleukin, *FSH* follicle-stimulating hormone, *LH* luteinizing hormone, *Iκκα* inhibitory κB-kinase alpha, *NF-κβ* nuclear factor-κβ, *IκB* inhibitory-κB, *IFN-*γ interferon-γ, *AR* aromatase enzyme, *A* androgens, *E* estrogens, *TGFβ* transforming growth factor β, *NOS* nitric oxide synthase

When discussing the influence of androgens on IL-1, it is important to consider both IL-1α and IL-1β. Zangeneh et al. performed a case–control study that involved a comparison between 85 cases (women with PCOS) and 86 controls (healthy women without PCOS). Specifically, the comparison involved the analysis of blood concentrations of IL-1α as well as other cytokines [11]. The researchers found that the women with PCOS had a mean IL-1α blood concentration of 401.40 pg/ml, whereas the control group had a mean of 19.32 pg/ml only [11], concluding that hyperandrogenism is positively correlated with increased production of IL-1α, which in turn inhibits estradiol secretion [11]. By opposing the action of estradiol, IL-1α consequently prevents normal development of follicles and repair of the endometrium after menstruation contributing to infertility typically seen in PCOS. The increased level of IL-1β then mediates both the synthesis of androgens and the blocking of effects of gonadotropins (Fig. 5) [34]. By countering the functions of gonadotropins, IL-1β impairs follicle development and ovulation in a way that complements the actions of IL-1α, contributing to infertility as noted above. Since hyperandrogenism results in increased production of M1 macrophages and neutrophils, these two cell types produce greater quantities of both IL-1α and IL-1β. Both of these interleukins then inhibit gonadotropin function to a greater extent, inhibiting the normal progression of the follicular and luteal phases of the ovarian cycle and contributing to PCOS-associated infertility [24, 25, 27].

Popovic et al. used an antagonist for the IL-1 receptor, anakinra, administered in 17 patients with PCOS; the patients were given 100 mg of anakinra daily for 28 days [30] after which CRP and **androgen levels were measured [34]. The researchers found that anakinra lowered CRP levels when measured on days 7, 21, and 28; it additionally led to a rise in estradiol concentration probably by removing the block on gonadotropin action [34]. Furthermore, five subjects with PCOS who were previously oligomenorrheic or amenorrheic showed menstrual bleeding [34]. However, anakinra did lead to a rise in androstenedione, testosterone, and DHT up until day 7 [34]. This suggests that anakinra has potential therapeutic benefits for some of the IL-1-induced symptoms of PCOS [34]. Based on this study, it appears that infertility in PCOS is directly related to the production of IL-1β with resultant inhibition of the gonadotropins FSH and LH.

### The role of Interleukin-6 (IL-6) and its signaling pathways in PCOS

Another interleukin that plays an important role in PCOS is IL-6 which can function by two signaling pathways. In the anti-inflammatory classical pathway, IL-6 binds to the IL-6 receptor (IL-6R) to form a complex with two molecules of glycoprotein 130 (gp130) [35]. In the pro-inflammatory trans-signaling pathway, IL-6 couples with IL-6R, which then attaches to gp130 in the membrane of a cell [35]. Both pathways can then result in the activation of the JAK/STAT pathway, MAPK pathway, or PI3K pathway [35]. Both the classical and trans-signaling pathways result in the activation of STAT3, which can then translocate to the nucleus to initiate transcription as part of the JAK/STAT pathway; in fact, this JAK/STAT process can then activate the MAPK and PI3K processes [35]. Regarding the MAPK pathway, the phosphorylated JAK can activate Ras by replacing the guanosine diphosphate (GDP) to which it is bound by guanosine triphosphate (GTP); this activated Ras consequently activates Raf-1, which activates MAPK so that it can translocate to the nucleus and begin gene transcription [35]. On the other hand, phosphorylated JAK can also induce the PI3K pathway by activating PI3K, the agent that then stimulates the conversion of phosphatidylinositol 4,5-bisphosphate (PIP2) into phosphatidylinositol (3,4,5)-trisphosphate (PIP3) and resultantly induces the PKB/AKT complex [35, 36]. Then, the next step is carried out by AKT, a serine/threonine-protein kinase that is alternatively known as protein kinase B (PKB); the key function of AKT is to induce the phosphorylation of proteins inside the cell to control multiple cellular activities ultimately [37]. In this particular pathway, AKT stimulates inhibitory κB-kinase alpha (Iκκα), which breaks down inhibitory-κB (IκB), an inhibitor protein; this removes the block on nuclear factor-κB (NF-κB) and allows it to enter the nucleus [35, 38, 39]. Once NF-κB enters the nucleus, it promotes the transcription of genes involved in inflammatory reactions [40]. Additionally, the activation of NF-κB is correlated with the formation of the NLRP3 protein that then binds to pro-Caspase-1 and ASC to form the NLRP3 inflammasome; this inflammasome then promotes the production of IL-1β and IL-18, ultimately contributing to the inflammatory state in PCOS [40]. NF-κB appears to be the key regulator of IL-6 levels since it can both stimulate and inhibit the production of IL-6, and NF-κB exerts this regulatory role by binding to its binding site on chromosome 7p21 or by influencing the activity of STAT3 [35].

As mentioned earlier, hyperandrogenism in PCOS promotes increased production of the IL-6-producing Th1 cells, neutrophils, and M1 macrophages; because of the resultant increase in IL-6 concentration, there is increased activation of the AKT pathway, which contributes to two features of PCOS [24, 25, 27]. First, it results in the formation of the NLRP3 inflammasome, which plays a role in PCOS-related inflammation [40]. Second, it results in increased growth and proliferation of adipocytes, which can lead to obesity seen in PCOS [41]. Studies have shown that PCOS is associated with increased levels of IL-6 that may contribute to insulin resistance [10]. As such, research is now being carried out to identify therapies that can manage PCOS by targeting and opposing IL-6 signaling. Currently, studies are investigating the possibility of targeting IL-6, IL-6R, gp130, or JAK. In addition, certain treatments like green tea, curcumin, and metformin have shown a decrease in IL-6 levels, so they may also be potential areas of nutraceutical exploration for treatment options [35], with inhibition of the JAK/STAT pathway that would downregulate the PI3K and MAPK pathways affecting all three signaling pathways used by IL-6.

Another important aspect of the role of IL-6 is its interrelationship with Cystatin-C. Recent research suggests a positive correlation of circulatory IL-6 and Cystatin-C concentrations; increased Cystatin-C levels predict an increased likelihood of cardiovascular disease, renal impairment and all-cause mortality. Thus, IL-6 may have a direct impact on the development of complications in PCOS, making it a potentially valuable target for reducing mortality in PCOS [42].

### The therapeutic potential of interleukin-22 (IL-22) in PCOS

The discussion of the interplay between PCOS and cytokines can be complemented by exploring the therapeutic effect of IL-22 and its ability to mediate the features of PCOS. Qi et al. investigated the benefits of IL-22 by studying three groups of mice: a dehydroepiandrosterone (DHEA) group given high levels of DHEA, a DHEA + IL-22 group given both DHEA and IL-22, and a control group with normal androgen levels [43]. In these groups, the mice that received DHEA were given 6 mg per 100 g of body weight for 21 days, while the mice receiving IL-22 were given 100 µg per kilogram of body weight for another 21 days [43]. The researchers summarized the effect of IL-22 into four key points. First, IL-22 reversed the metabolic dysfunction in PCOS by leading to reduced glucose levels in the glucose tolerance test, as well as restoring normal insulin responsiveness in the insulin tolerance test [43]. Second, the administration of IL-22 also countered hormone disturbances by reducing both the testosterone levels and the LH/FSH ratio, both of which are high in women with PCOS [43]. Third, IL-22 was also associated with improved ovarian function, which can be seen in how the mice who received IL-22 had improved numbers of corpora lutea, enhanced production of embryos, and fewer cystic follicles [43]. Finally, IL-22 also contributed to the browning of white adipose tissue, which plays a role in reversing glucose metabolism dysfunction [43]. The finding regarding the interaction between IL-22 and brown adipose tissue can also be further explained by considering that the key function of brown adipose tissue is heat generation. In order to produce heat, there would be an increased breakdown of glucose and a resulting decrease in glucose levels when measured by the glucose tolerance test, therefore explaining how IL-22 can improve glucose metabolism.

### Interferon-γ and its effect on fertility in PCOS

Another key cytokine involved in PCOS pathogenesis is IFN-γ, which appears to be associated with leptin. Wang et al. sought to identify the role of leptin in the development of PCOS and showed that women with PCOS had a higher level of leptin than the control subjects [44]. Wang et al. also found a positive correlation between the increased amount of leptin and the homeostatic model assessment for insulin resistance (HOMA-IR); additionally, this research also established the relationship between HOMA-IR and the Th1-controlled (T helper type 1-controlled) inflammatory response which produces IFN-γ [44, 45]. So, by combining these two statements, it seems that leptin excess in PCOS is directly positively correlated with increased IFN-γ levels [44]. As a result, Wang et al. concluded that IFN-γ had increased production in women with PCOS under the influence of leptin [44]. What is interesting to note is that based on these findings, the production of IFN-γ may contribute to the production of excess androgen in PCOS; by stimulating granulosa cell apoptosis, IFN-γ eliminates the aromatase enzyme in these cells and therefore ensures that the steroid synthesis pathway ends at androgen rather than estrogen.

Since hyperandrogenism increases the production of Th1 cells, it also contributes to the increased production of IFN-γ by these cells [24]. This excess concentration of IFN-γ has several effects in relation to PCOS. First, it leads to the apoptosis of granulosa cells, which results in the termination of the follicle. Second, by eliminating granulosa cells, IFN-γ also inhibits the aromatase enzyme within these cells, which normally converts androgens to estrogens. Therefore, without the proper function of this enzyme, estrogen production is inhibited, and it is no longer able to effectively promote the growth of the follicles and repair the endometrium after menstruation. Both of these effects are associated with infertility in PCOS [44].

### The multiple effects of transforming growth factor-β (TGFβ) in the pathogenesis of PCOS

Another key cytokine involved in PCOS is TGFβ, whose dysfunctional signaling pathway appears to be associated with the development of PCOS. In normal signaling, secreted TGFβ is bound to both its propeptide and latent TGFβ binding proteins (LTBP); this complex then binds to either fibrillin or follistatin and remains inactive until it is needed. Once required, it dissociates from the complex with fibrillin or follistatin and binds to a TGFβ type II receptor, phosphorylating a type I receptor. This results in the phosphorylation of a receptor-regulated SMAD, a protein inside the cell, which binds to a coSMAD to eventually enter the nucleus and alter gene transcription [46, 47].

Because fibrillin 3 is the key fibrillin binding to TGFβ to keep it in an inactive state, numerous studies have tried to investigate whether there is a relationship between the fibrillin 3 gene and fetal development of PCOS. If fibrillin 3 is defective, TGFβ will no longer be complexed in an inactive state and will instead activate fibroblast activity, leading to excessive amounts of collagen and stroma. When investigating the association between fibrillin 3 expression and PCOS, one study concluded that “since fibrillins are stromal matrices and since the ovarian stromal compartments are altered in women with PCOS, fibrillin 3 expression in the developing fetal ovary, via the activity of TGFβ to regulate stromal formation and function, could predispose an individual to PCOS in later life” [48]. Another study conducted on monkeys with PCOS found that many of the genes involved in TGFβ signaling were repressed due to having methylated promoters [46]. Therefore, both these studies suggest that having genetic abnormalities contributes to abnormal TGFβ signaling that is associated with fetal development of PCOS.

TGFβ dysregulation may lead to the development of excessive amounts of collagen and stroma. Additionally, neutrophilia in PCOS could result in increased production of TGFβ, which would also have the same influence on collagen and stroma. Either way, this disruption may lead to two main effects. First, it can result in the production of abnormally high quantities of androgen, leading to the hyperandrogenism pathognomonic of PCOS [46]. Second, the excessive stroma may prevent the follicle from increasing in size and developing, which may contribute to a failure in ovulation and PCOS-related infertility [46].

Abnormal TGFβ signaling, or excessive production due to neutrophilia, can affect cardiovascular and metabolic function in PCOS. Excessive activity of TGFβ may stimulate smooth muscle cells in blood vessel walls to produce collagen, which is associated with an increased risk of atherosclerosis [46]. TGFβ may also induce endothelial cells to produce IL-6, which can contribute to obesity [46]. TGFβ may contribute to hypertension by inhibiting nitric oxide synthase and promoting the synthesis of endothelin I, both of which ultimately result in vessel constriction [46]. TGFβ dysfunction can also activate the renin–angiotensin–aldosterone system (RAAS), which is associated with increased risk of atherosclerosis and diminished arterial compliance [46]; through stimulating RAAS, TGFβ may also be associated with hypernatremia in PCOS due to actions of angiotensin II and aldosterone.

To summarize the relationship between TGFβ and PCOS, it appears that hyperandrogenism contributes to high levels of neutrophils which in turn produce large quantities of TGFβ [10]. This high concentration of TGFβ then contributes to hypertension seen in PCOS by inducing endothelin I, inhibiting NO, and activating RAAS [46].

### Conflicting findings regarding cytokines in PCOS

Even though the previously discussed research provided evidence for an association between the various cytokines and PCOS, other studies contradict the findings. In a meta-analysis, no significant association between the TNF-ɑ and IL-1β polymorphisms and the development of PCOS was found. Regarding IL-6, the researchers only found an association between the IL-6 polymorphism and PCOS development in a homozygote comparison and allelic model [49]. These data highlight the need for further definitive and robust studies on the role of cytokines in PCOS.

The disagreement regarding the interaction between androgen and cytokines can be explained in two ways. First, many of the studies suggesting the presence of a positive correlation between hyperandrogenism and cytokine activity were carried out on rats, so it may be the case that these physiological interactions occur differently in humans. Second, one interpretation of all the findings presented so far may be that they do not actually contradict each other and may coexist in the following way: even though the interactions between TNF-ɑ or IL-1β and PCOS may not be genetic in origin, they may arise during the life of the female; in other words, the association may be acquired rather than hereditary.

## The effect of androgens on signaling pathways in PCOS

**Fig. 6:**
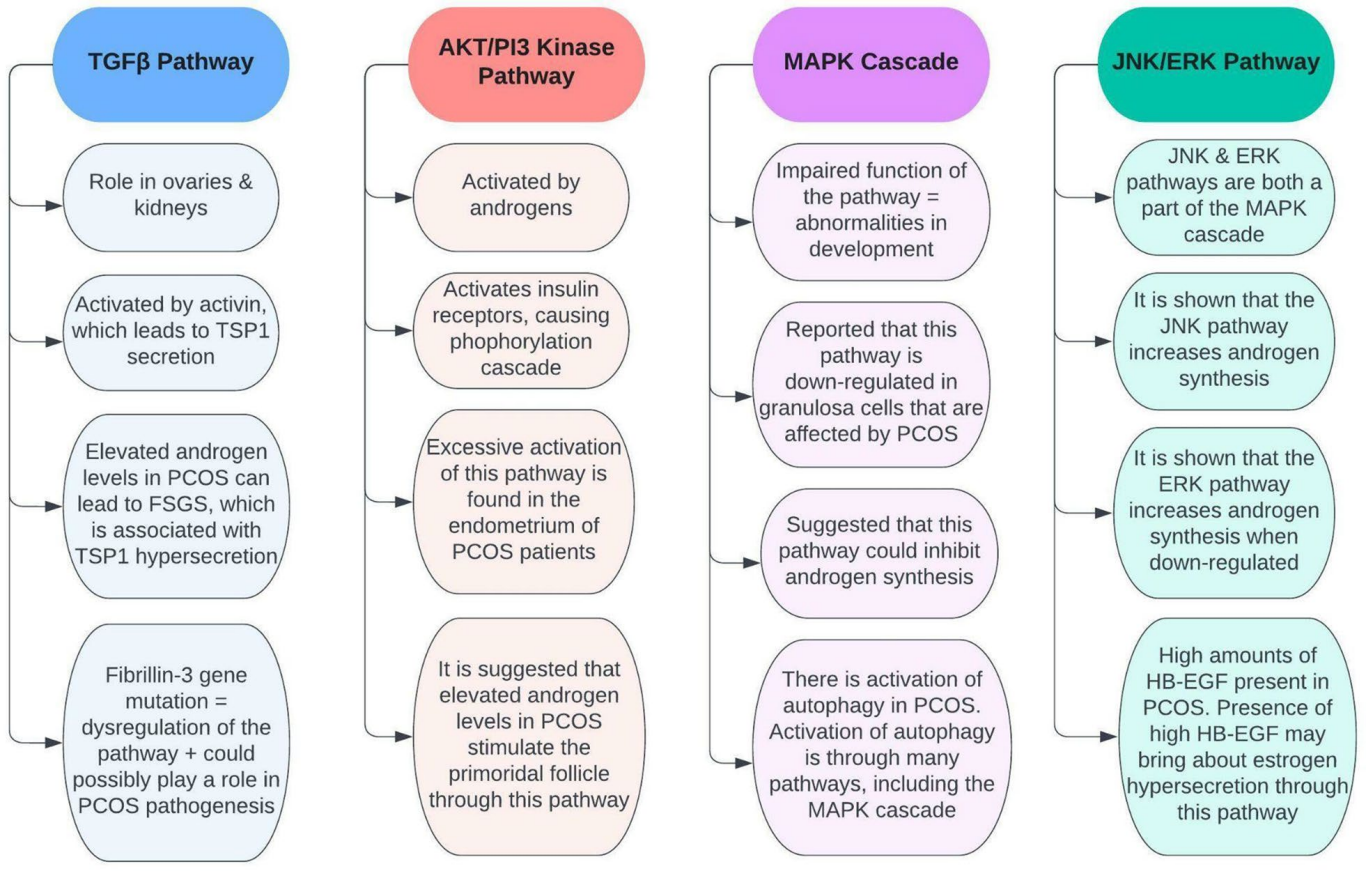
The major pathways known to be involved in PCOS. *TSP1* thrombospondin 1, *FSGS* focal segmental glomerulosclerosis, *HB-EGF* heparin-binding EGF-like growth factor

Studies have shown numerous connections between PCOS and several pathways that occur in the body. These pathways include the TGFβ pathway, the AKT/PI3 kinase pathway, the MAPK cascade, and the JNK/ERK pathway (Fig. 6). For example, TGFβ pathway dysregulation has been suggested to have a role in PCOS pathogenesis [50], while the AKT/PI3 kinase pathway seems to be overactivated in the endometrium of PCOS patients [13]. Furthermore, the MAPK cascade is suggested to be down-regulated in PCOS [14], and the JNK/ERK pathway has some control over androgen synthesis [15]. Overall, gaining a comprehensive understanding of how these pathways are associated with PCOS could give insight into how to control the condition more effectively.

### The transforming growth factor-β (TGFβ) pathway in PCOS

The TGFβ signaling pathway is involved in several processes and has a crucial role in the ovaries and kidneys. Activin activates this pathway, which in turn results in the release of the hormone FSH from the ovaries. In the kidneys, activin will lead to the secretion of thrombospondin 1 (TSP1), which has a role in apoptosis and cell growth [51]. In PCOS, the individual has elevated androgen levels. This can lead to the damage of the androgen receptors that are located in the kidney, which in turn can result in a number of renal disorders, such as focal segmental glomerulosclerosis (FSGS) [51]. Elevated androgens damage podocytes in the kidneys and lead to TSP1 hypersecretion, which is associated with FSGS. Both PCOS and FSGS are intertwined as they are both associated with the TGFβ signaling pathway [12].

Recent evidence has shown that PCOS is connected with the fibrillin-3 gene. Fibrillins and several other molecules regulate the TGFβ pathway. Mutations in this gene can give rise to TGFβ dysregulation, which could possibly play a role in the pathogenesis of PCOS [50]. Dysregulation of this pathway results in cardiovascular, metabolic, and reproductive issues. Cardiovascular problems include inflammation and the hyperactivity of RAAS. Metabolic issues involve insulin resistance, impaired glucose tolerance, and type 2 diabetes. Reproductive issues, on the other hand, include the abnormal secretion of gonadotropin, as well as hyperandrogenism [46]. However, there is not enough evidence to support that TGFβ dysregulation is involved in the pathogenesis of PCOS. Further research is required, as the TGFβ pathway and PCOS are closely associated with one another [46].

### The phosphatidylinositol 3-kinase (AKT/PI3) pathway in PCOS

The AKT/PI3 kinase pathway is associated with the insulin signaling pathway. Once insulin receptors are activated, a phosphorylation cascade occurs. This cascade commences due to the autophosphorylation of the receptors and by insulin receptor substrate proteins (such as IRS-1 and IRS-2) activating. The substrate proteins will then induce PI3 kinase to phosphorylate PIP2 to PIP3 [32]. This pathway is involved in cell division, cell growth, survival, metabolism, etc. [52].

**Fig. 7:**
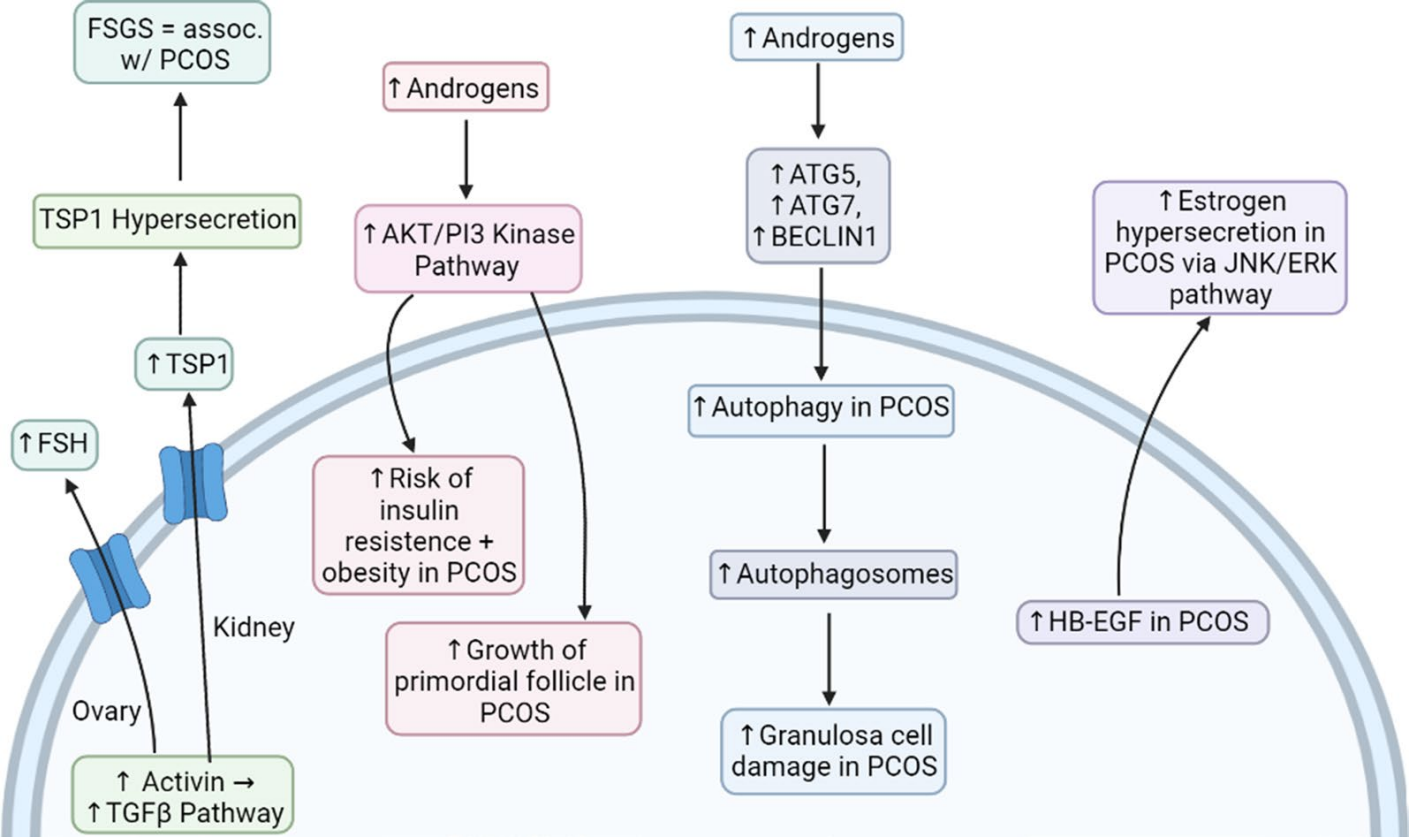
The signaling pathways involved in the development of complications in PCOS. *FSH* follicle-stimulating hormone, *TSP1* thrombospondin 1, *FSGS* focal segmental glomerulosclerosis, *HB-EGF* heparin-binding EGF-like growth factor

A study was conducted in order to see the activation of the AKT/PI3 kinase pathway in the endometrium in women who have PCOS. The PCOS participants were divided into two groups: insulin-resistant and non-insulin-resistant. The results of the study revealed that the expression of p-AKT (phosphorylated AKT) was lower in the non-insulin resistance group compared to the insulin-resistant group, which had higher p-AKT expression. The study concluded that there was excessive activation of the AKT/PI3 kinase pathway in the endometrium of PCOS patients [13]. This overactivation of the pathway may be involved with endometrial hyperplasia, as well as carcinoma. The presence of endometrial carcinoma can be investigated by tissue testing for exosomes carrying microsomal RNA (miRNA), such as miRNA-21 which is a valuable biomarker even in other malignancies such as melanoma [53, 54]. The treatment and disease course of endometrial carcinoma can be investigated using endometrial organoids, which are *in-vitro* tissue models [55]. Additionally, the overactivation of this pathway in the endometrium may result in the risk of insulin resistance and obesity in people with PCOS (Fig. 7) [13]. The AKT/PI3 kinase pathway is activated by androgens, such as estradiol [56, 57]. Since there are elevated androgen levels in PCOS patients, this would most likely result in the overactivation of the pathway. This implies that hyperandrogenism from PCOS directly affects the AKT/PI3 kinase pathway.

Studies suggest that elevated androgen levels in PCOS stimulate the activation of the primordial follicle through the AKT/PI3 kinase pathway. This is because testosterone activates the AKT/PI3 kinase pathway, which in turn activates the primordial follicle [50]. In addition, it is hypothesized that hyperandrogenism in PCOS results in increased growth of the follicles via upregulation of insulin-like growth factor (IGF). IGFs stimulate the development of the follicles and are commonly elevated in PCOS, and it is suggested that elevated IGFs are a possible cause of the accelerated growth of the follicles in the ovaries of PCOS women [58]. Furthermore, hyperandrogenism has been shown to diminish the expression of the Cx43 protein in granulosa cells. This suggests that excess androgens in PCOS could possibly hinder how granulosa cells communicate, leading to dysfunction in ovulation, as well as developmental dysfunction of the follicles [58].

### The Mitogen‑activated protein kinase (MAPK) Cascade in PCOS

The MAPK cascade is a series of pathways that regulate several processes, such as apoptosis, cell division, and differentiation, as well as stress responses. Impaired function of the MAPK cascade can result in abnormalities in development and is also associated with the progression of numerous medical conditions [59].

A study reported that the MAPK cascade is down-regulated in granulosa cells that are affected by PCOS. This would, of course, affect the functions of the granulosa cells. Through a study, it has been shown that the inhibition of the MAPK cascade in granulosa cells results in the expression of the CYP17 enzyme [14]. This enzyme is involved in the synthesis of androgens [60]. In addition, a majority of studies have shown that the expression of the StAR protein is increased in PCOS [14]. The StAR protein is also involved in androgen synthesis [61]. These studies suggest that the MAPK cascade could reduce the expression of the StAR protein and the CYP17 enzyme, which would inhibit androgen synthesis [14]. However, there are a few opposing studies, and further research is needed for clarification [14].

A study has shown that there is an activation of autophagy in PCOS [14]. Autophagy is the body’s process of removing damaged cells, organelles, pathogens, etc. It plays an important role in development [62]. The activation of autophagy is through several pathways, one of which is the MAPK cascade. A possible cause of granulosa cell apoptosis is the accumulation of autophagosomes. Hyperandrogenism in PCOS could result in the expression of several genes that induce autophagy, such as ATG5, ATG7, and BECLIN1. However, there is still a dispute on the activation of autophagy in PCOS, so further research is required [14]. This controversy aside, it is possible that excess androgens in PCOS can accelerate the process of autophagy, thus increasing amounts of accumulated autophagosomes. Elevated androgen levels could therefore cause apoptosis of granulosa cells.

### The c-Jun N-terminal kinase (JNK)/extracellular signal-regulated kinase (ERK) Pathway in PCOS

The JNK and ERK pathways are both a part of the pathways involved in the MAPK cascade [63]. They aid in the regulation of apoptosis and are also involved in many cellular processes [64]. One of the major enzymes in androgen synthesis is cytochrome p450 17ɑ-hydroxylase (P450c17). In PCOS women, this enzyme’s activities are quite high, which is one of the major causes of hyperandrogenism in PCOS. Therefore, changes in this enzyme will affect androgen production [15]. While stated earlier in a study that the MAPK cascade is down-regulated in PCOS, other findings have found it not to be that simple. Other studies have shown that the JNK pathway (which is a part of the MAPK cascade) is amplified in PCOS. Also, some studies have shown that the ERK pathway specifically could increase androgen synthesis when down-regulated. Furthermore, findings have shown that the JNK pathway is involved in the stimulation of P450c17 and could possibly explain the elevated androgen levels in PCOS since this pathway is enhanced in this condition [15]. It can be deduced that not all the pathways of the MAPK cascade function the same way. The JNK pathway has been shown to increase androgen synthesis, while the ERK pathway only increases androgen synthesis when it is reduced. It can be concluded that the ERK pathway is down-regulated in PCOS, therefore contributing to elevated androgen levels. On the other hand, the JNK pathway may not be down-regulated in PCOS, which results in androgen synthesis, therefore, possibly contributing to hyperandrogenism as well.

Heparin-binding EGF-like growth factor (HB-EGF) is a glycoprotein that plays a role in the female reproductive system, as well as in the development of the embryo. A recent study showed that copious amounts of HB-EGF are present in the follicular fluid in PCOS. It is suggested that the presence of abundant HB-EGF in the fluid may bring about estrogen hypersecretion through the JNK/ERK pathway, resulting in apoptosis of granulosa cells and mitochondrial dysfunction [65]. As estrogen is considered to be an androgen, the influence of HB-EGF on the JNK/ERK pathway may be another contributor to hyperandrogenism in PCOS.

## Androgen effects on adipocytes and steroid production in PCOS

**Fig. 8:**
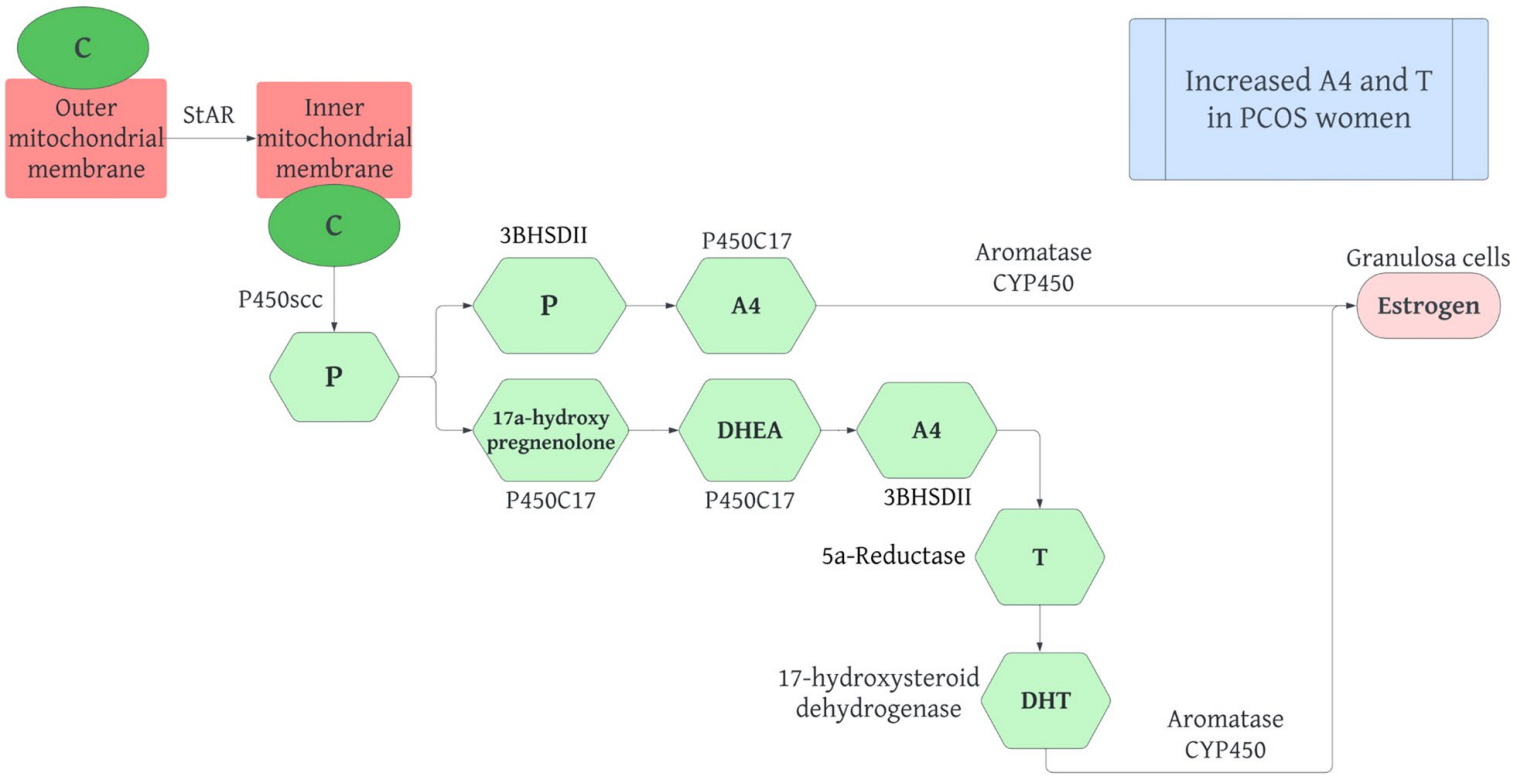
The normal steroidogenesis pathway in humans. *C* Cholesterol, *StAr* Steroidogenic Acute Regulatory Protein, *P450scc* Cholesterol side-chain cleavage enzyme,* P* Progesterone,* 3* β *HSD II* Type II 3-hydroxysteroid-5-steroid dehydrogenase, *P450C17* Cytochrome p450 17ɑ-hydroxylase, *A4* Androstenedione, *DHEA* Dehydroepiandrosterone,* T* Testosterone, *DHT* 5ɑ-dihydrotestosterone

Androgens are produced by adipose tissue in the ovaries and the adrenal glands from the common precursor, cholesterol. Cholesterol, in turn, allows for the steroidogenic pathway to occur. Hence, understanding the regular synthesis of steroids in humans is important (Fig. 8). The steroidogenic pathway involves several proteins and enzymes producing active steroid hormones [66]. Cholesterol in the outer mitochondrial membrane is translocated to the inner mitochondrial membrane by steroidogenic acute regulatory protein (StAR). Next, cholesterol is converted into pregnenolone by cytochrome p450 side chain cleavage, encoded by the CYP11A gene, in a rate limiting reaction. Pregnenolone can then be hydroxylated into 17ɑ-hydroxypregnenolone by P450C17, which can also use lyase activity to convert 17ɑ-hydroxypregnenolone into DHEA. P450C17 is found in the theca cells of the ovaries and is significant in determining whether androgens or progestins are produced from the steroidogenic pathway. Pregnenolone can also be converted into progesterone by type II 3 $\beta$-hydroxysteroid-${\Delta }^{5}$-steroid dehydrogenase (3$\beta$HSDII). Progesterone can be converted into androstenedione (A4) by P450C17. A4 can also be produced by DHEA through 3 $\beta$ HSDII which is then converted into testosterone (T) by 17 $\beta$-hydroxysteroid dehydrogenase. These reactions occur in ovarian theca cells and allow for A4 and T to be taken up by granulosa cells. In granulosa cells A4 and T are converted into estrogen by the aromatase cytochrome P450 enzyme, which is controlled by FSH. T can be converted into DHT by 5ɑ-reductase in peripheral tissues [58]. Several studies suggest that women with PCOS display elevated A4 and T levels [14].

In PCOS, androgens have deleterious impacts on adipose tissue function. The influence of androgens on adipocyte function is determined by levels of circulating androgen, “androgen receptor density and affinity,” [16] and the enzymatic activity of local steroids. The ability of adipocytes to modulate steroidogenic enzyme activity is of particular importance in the progression of hyperandrogenism in PCOS due to the conversion of weak androgens to strong androgens. For this reason, the interrelation of hyperandrogenism and obesity in women with PCOS may originate from increased adipocyte steroidogenesis. With regards to androgen receptors, which are present on preadipocytes as well as mature adipocytes, androgens can affect gene expression, cell differentiation, and proliferation, “and carbohydrate and lipid metabolism” [16]. Androgens also increase the transcription of androgen receptors on mononuclear cells allowing for the release of adipocytokines, and the concentration of adipocytokines is further increased through production by immune cells. Adipocytokines, in turn, stimulate the secretion of steroidogenic cells. This is seen in hyperandrogenism in PCOS, which allows for the abnormal secretion of adipocytokines.

Compelling studies indicate that abnormalities in steroidogenic cells in theca cells of the ovaries can result in the overproduction of androgens in women with PCOS. De Mederios et al. published a review in 2021 regarding the “interrelationship between adipocytes and adrenal and ovarian steroidogenic cells in animal models and humans with or without PCOS” [17]. Utilizing articles published in PubMed in both the English and Portuguese languages, the researchers found that “glucocorticoids and sex steroids” [17] regulate the differentiation and function of adipocytes resulting in dysfunctional adipose tissue in both animals and women. The study outlined that “women with PCOS secrete unbalanced levels of adipocytes,” specifically increased amounts of leptin and decreased amounts of adiponectin [17]. “Leptin positively correlates with body mass index, waist hip ratio, levels of total cholesterol, triglyceride, LH, estrogen, and androgens” [17]. As previously mentioned, excess leptin is also positively correlated with IFN-γ. IFN-γ controls the enzymatic activity of 17ɑ-hydroxylase and aromatase in granulosa cells which results in the steroidogenesis pathway ending with increased production of androgens rather than estrogens. This association between leptin and IFN-γ reinforces the interplay of PCOS, androgens, and the immune system [44]. “Adiponectin negatively correlates with body mass index, waist hip ratio, glucose, insulin, triglycerides, and androgens” [17]. Androgen production is decreased through cholesterol side chain enzyme, 17ɑ-hydroxylase, LH receptors, and StAR.^17^ There is a positive correlation between resistin and testosterone, the expression of 17ɑ-hydroxylase in theca cells, and body mass index [17].

The 2022 study conducted by De Medeiros et al. [16] reviewed published studies from 1983 to 2021. The researchers highlighted that androgens on androgen receptors act as transcription factors that modulate gene expression, which is controlled by several factors, including “peroxisome proliferator activated receptor gamma and the mitogen activated protein kinase cascade” [16]. The researchers conveyed that androgens repress the differentiation of adipocytes epigenetically through lowered transcription of peroxisome proliferator activated receptor gamma. Consequently, inhibition of preadipocyte differentiation and maturation is modulated by T and DHT in subcutaneous and visceral adipose tissue depots. Androgen receptors also suppress IL-12ɑ expression to decrease NK cell efficiency. Increased levels of T also encourage adipocyte hypertrophy and the excessive production of adipocytokines. In women with and without PCOS, increased amounts of T inspire lipogenesis; however, there is impaired lipolysis in non-obese women with PCOS. In terms of abnormal differentiation and proliferation of adipose tissue, fat tissue is rearranged from subcutaneous to visceral depots. As a result, hyperandrogenism in women with PCOS leads to greater centralized adiposity. This causes an increased amount of fat localizing in the trunk in obese and normal-weight women with PCOS [14].

Abnormalities in the steroid synthesis pathway can contribute to the impaired activity of immune cells and hence irregularities in adipocytokine secretion, as discussed previously. To summarize, increased T concentrations are associated with normal FOR, which allows for increased immune system activation and the production of APF. Increased production of androgens causes the conversion of macrophages to the M1 state, which allows for increased release of cytokines, neutrophilia, and increased monocyte production. Androgens also influence mast cells to synthesize IL-33 leading to the production of basophils and innate lymphoid cells [8]. Increased androgen concentrations also reduce IL-12ɑ levels, and consequently, fewer NK cells are recruited, which can lead to a cytokine disorder. Studies have shown that increased androgen administration promotes TGFβ production, which inhibits IL-7 and results in the inhibition of B cell synthesis [8]. Androgens have an inhibitory effect on DCs, causing a reduction in the production of cytokines produced by them. Contrarily, androgens are associated with decreased eosinophil production, and excess DHT production seems to be associated with peripheral T cell death [8]. Additionally, increased estrogen levels lead to excess inflammatory cytokine secretion from Th1 cells. These conclusions again emphasize the complex relationship between androgens, immune cells, and the production of adipocytokines in PCOS.

Hence, androgens exert damaging effects on adipocytes. In women with PCOS, adipocytes produce androgens, and androgens regulate adipocyte differentiation, proliferation, adipogenesis, hypertrophy, and most importantly, androgens stimulate the secretion of steroidogenic cells and adipocytes [16]. Women with PCOS secrete increased levels of leptin and resistin and decreased amounts of adiponectin. Additionally, irregularities in steroidogenesis can lead to impaired immune cell activity and dysfunctional adipocytokine secretion. These regulatory functions of androgens on adipocytes and adipocytokines can worsen the symptoms and effects of PCOS.

## Hyperandrogenism, insulin resistance, and obesity in PCOS

### Hyperandrogenism in PCOS

PCOS in women is characterized by hyperandrogenism (HR). HR is defined as an increase in levels of androgens in females caused by irregularities in ovarian or adrenal functions. Clinical manifestations of HR in PCOS can include acne, hirsutism, alopecia, weight gain, amenorrhea, and insulin resistance [34]. As mentioned before, this symptom associated with PCOS in women often involves abnormalities in steroid synthesis. “Recent studies also indicate that the hyperandrogenic phenotype in PCOS is familial” [66]. This suggests that genes, particularly those involved in the steroid synthesis pathway, can be maternally inherited to cause HR and PCOS. It has also been hypothesized that fetal exposure to excess androgens can result in increased secretion of LH, changes in differentiation of thecal cells, and a male-like arrangement of fat in females. Additionally, experimental studies have outlined that IL-1, a proinflammatory cytokine that induces androgen production in the ovaries and abnormalities in gonadotropin signaling, plays a role in disrupting fertility [34].

A review article by Ashraf et al. highlighted that the primary effect of excessive androgen in women is abnormalities in folliculogenesis. In women without PCOS, gonadotropin-releasing hormone (GnRH) normally is secreted by the hypothalamus, which triggers the release of gonadotropins, LH and FSH, by the pituitary gland. LH acts on theca cells of the ovaries to produce androgens. FSH acts on granulosa cells of the ovaries to convert androgens to estrogens, most significantly, estradiol to develop follicles. In women with PCOS, there is “an imbalance in the hypothalamic-pituitary-ovarian axis,” which results in increased gonadotropin production, particularly increased LH over FSH, which increases the “LH/FSH ratio in PCOS” [66]. An increase in LH drives high androgen production by theca cells, and decreased FSH results in androgen accumulation, and hence HR, due to less conversion of androgen to estrogen. Due to this neuroendocrine change in women with PCOS, several follicles are arrested in the “preantral and antral stages” of folliculogenesis [66]. This leads to hyperplasia of theca cells and an increase in the follicular fluid, which forms cyst-like structures along the periphery of the ovaries [66]. Hence, increased LH production drives increased androgen production in HR. With regards to IL-1, which inhibits the actions of estrogen, the cytokine counters the regular development of follicles and repair of the endometrium wall after menstruation, which can lead to infertility. It was previously discussed that IL-1β production is increased through HR caused by DHT excess [9], resulting in increased production of androgens and inhibition of gonadotropins [34]; this further reinforces infertility in PCOS women. HR can also increase the synthesis of IL-1α, which can cause increased fertility issues in PCOS as well.

**Fig. 9:**
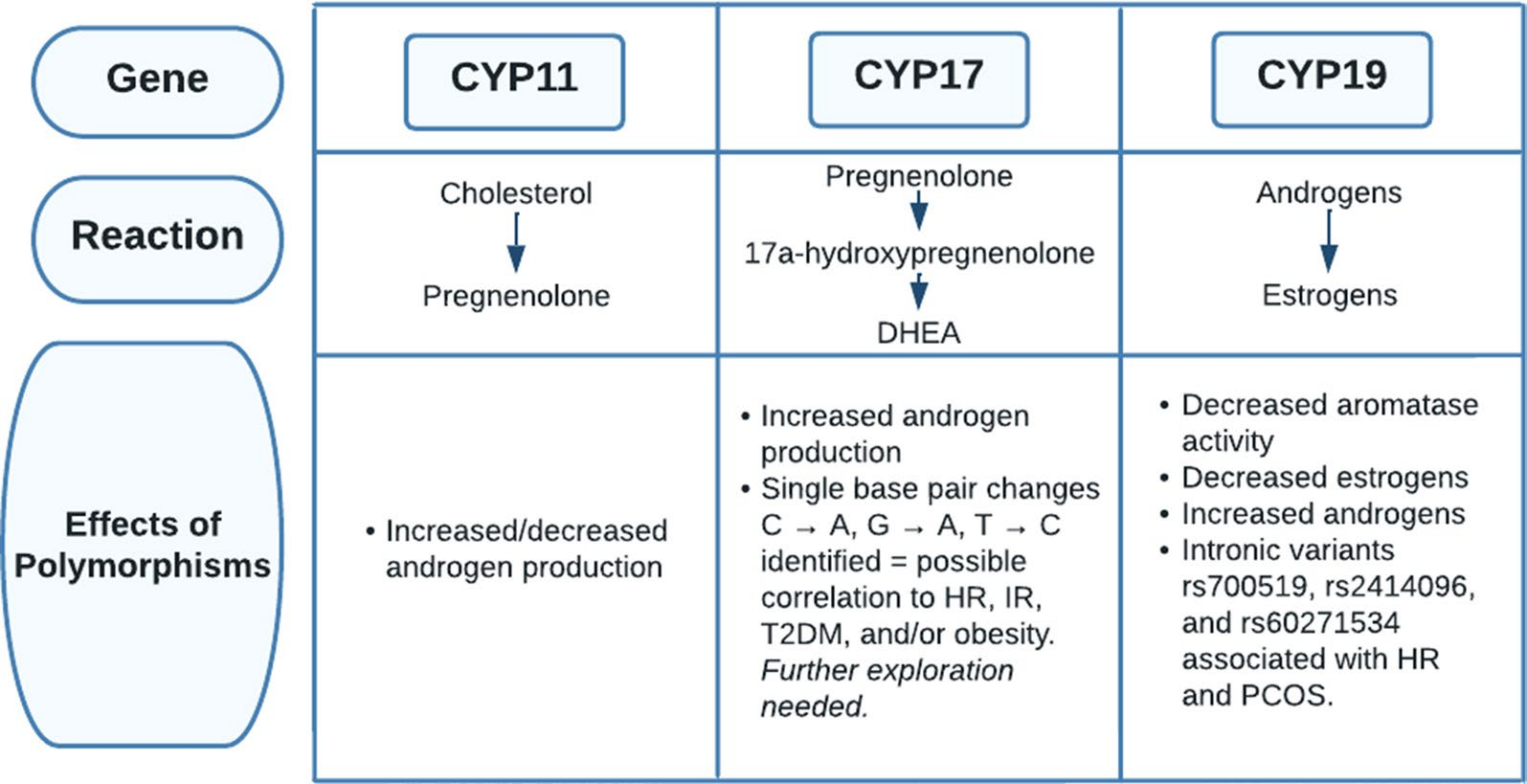
An outline of the effects of polymorphisms in CYP genes on the steroidogenesis pathway in PCOS patients. *DHEA* dehydroepiandrosterone,* C* cytosine, *A* adenine, *G* guanine,* T* thymine, *HR* hyperandrogenism, *IR* insulin resistance, *T2DM* type 2 diabetes mellitus

The same article also studied the relationship between genes involved in the steroid synthesis pathway and HR in women with PCOS. The first step in steroidogenesis, the conversion of cholesterol into pregnenolone, is catalyzed by the cytochrome side-chain cleavage enzyme, which is encoded by the CYP11 gene (Fig. 9). Several studies suggested that polymorphisms in the CYP11 gene can result in the upregulation or downregulation of CYP11, leading to increased or decreased androgen production [66]. Next, the CYP17 gene encodes for the cytochrome P450 17ɑ-hydroxylase-17, 20-lyase enzyme, which converts pregnenolone to 17ɑ-hydroxypregnenolone and 17ɑ-hydroxypregnenolone into DHEA [66]. Several studies have highlighted that dysregulation of the CYP17 gene is associated with increased androgen production in PCOS. Crocitto et al. outlined a single base pair change from C to A in the CYP17 gene on nucleotide 5471 at intron 6 [67]. Miyoshi et al. identified a G to A transition in the CYP17 gene on nucleotide 1951 in the promoter region [68]. The most significant single base pair change was noted by Carey et al., where there was a transition of T to C in the 5’ untranslated region [69]. These three studies identified an association between single nucleotide polymorphisms and PCOS. Other studies have displayed that some polymorphisms are associated with PCOS while others are not. Studies in India concluded that a polymorphism in the promoter -34 region of the CYP17 gene had no relation to HR, centralized obesity, or insulin resistance [70, 71]. Lastly, the CYP19 gene encodes for the aromatase P450 enzyme, which converts androgens into estrogens. The cytokine IFN-γ allows for apoptosis of granulosa cells and follicle termination. The apoptosis of granulosa cells causes IFN-γ to inhibit the aromatase enzyme as well, and this results in the inhibition of estrogen production [44]. Several patients with HR showcase decreased aromatase activity. Erickson et al. highlighted diminished aromatase activity in granulosa cells in women with PCOS [72]. Jakimiuk et al. demonstrated that there is decreased aromatase expression and lowered levels of aromatase P450 mRNA in PCOS follicles, resulting in decreased levels of estradiol [73]. These studies indicate that diminished aromatase activity can be associated with increased levels of androgen, leading to impaired development of follicles. A study by Chen and colleagues reported a decrease in the ratio of estrogen to testosterone in women with PCOS and that there is an increased risk of developing PCOS associated with the “intronic variant rs2414096” of the CYP19 gene [74]. Similarly, several other studies concluded that rs700519, rs2414096, and rs60271534 of the CYP19 gene are all associated with PCOS and HR due to reduced aromatase activity [66].

Thus, hyperandrogenemia can contribute to the abnormal manifestations of enzymes involved in steroidogenesis and increased gonadotropin production through the interference of feedback of the hypothalamus and pituitary gland by androgens. However, gonadotropin production can also be inhibited by the IL-1β cytokine, causing infertility [34]. Increased LH leads to hyperplasia of theca cells resulting in increased steroidogenesis and androgen production. The CYP11, CYP17, and CYP19 genes that encode for enzymes in the pathway of steroid synthesis increase 17-hydroxyprogesterone, testosterone, and androstenedione but reduce aromatase activity [66]. Aromatase activity is also decreased through the cytokine IFN-γ [44]. Overall, these genes and the IFN-γ cytokine increase androgen production.

### Insulin resistance in PCOS

Insulin resistance (IR) and, consequently, hyperinsulinemia are also characteristic features of PCOS. IR in PCOS is typically manifested as centralized adiposity and obesity. IR in PCOS can be caused by several factors. In at least 50% of IR cases in women with PCOS [75], there is serine phosphorylation by phosphorylation-172–1-receptor substratum [76], which inhibits the insulin receptor signal [75]. Since serine phosphorylation regulates the activity of cytochrome p450 17ɑ-hydroxylase, an enzyme involved in androgen synthesis, it can be said that a single defect in this enzyme can cause both IR and hyperandrogenism in PCOS. A genetic mutation of MTNR1B in PCOS can also delay the production of insulin resulting in rapidly increased blood glucose levels [76]. 1,25-dihydroxyvitamin D plays a role in glucose homeostasis, where it improves the insulin sensitivity of liver cells, skeletal muscle, and adipose tissue and improves beta-cell function. IR and T2DM are associated with impaired beta cell function; hence, insufficient vitamin D can cause IR in PCOS as well [76]. Additionally, increases in the proinflammatory cytokine TNF-ɑ also inhibit the tyrosine kinase function of the insulin receptor, further causing IR in PCOS [33]. Lastly, decreases in nitric oxide (NO) and increases in endothelin1 can cause IR in endothelial artery cells, leading to the synthesis of vasoconstrictors and reduced vasodilation by insulin in PCOS-positive women [76]. Dysregulation of the cytokine TGFβ leads to excessive production of collagen and stroma which consequently results in hypertension through the inhibition of NO and activation of RAAS. The development of hypertension through this mechanism causes impaired vasodilation and increased vasoconstriction, which leads to alterations in glucose uptake [46].

In a study of IR in women with PCOS, Ding et al. determined that IR in PCOS is not related to weight; nevertheless, it can be worsened by obesity [76]. When women with PCOS were compared to weight-matched reproductively healthy women, insulin-regulated glucose clearance was reduced by 35–40% in women with PCOS [76]. However, hepatic IR, which is specified by increased post absorptive glucose production and diminished suppression of endogenous glucose production by insulin in hepatocytes, is only seen in obese women with PCOS [76]. Women with PCOS also seem to showcase increased baseline insulin amounts but decreased insulin responsiveness to carbohydrates. Next, it is important to discuss the disposition index. Normally, it is when changes in insulin sensitivity result in subsequent changes in the secretion of insulin in order to regulate a normal glucose tolerance [76]. In women with PCOS, the disposition index is decreased in comparison to weight-matched reproductively healthy women. Further, mobilization of adipose tissue through IR increases levels of plasma-free fatty acids, which reciprocally induces IR by inactivating enzymes that modulate glucose transport, such as dehydrogenase pyruvate. A recent study recruited 45 obese PCOS patients in Italy to undergo treatment with acetyl-l-carnitine, l-carnitine, l-arginine, and *N*-acetyl-cysteine for up to 24 weeks [77]. Results showed that after 12 and 24 weeks of administration, all patients had decreased plasma insulin, triglyceride, and total cholesterol levels but increased high-density lipoprotein. Hyperinsulinaemic PCOS patients showed an improved hepatic insulin extraction index [77]. These results hence highlight that hyperinsulinaemic PCOS patients have impaired liver functions, and treatment with acetyl-l-carnitine, l-carnitine, l-arginine, and *N*-acetyl-cysteine decreases plasma insulin and thus can manage hyperinsulinemia.

The study conducted by Ding et al. also explained that there might be a “mutual act of IR and hyperandrogenemia on the ovaries and adrenal glands,” thus resulting in PCOS [76]. Insulin allows for ovarian androgen production and increases the levels of free testosterone, which results in the inhibition of SHBG and consequently plays a role in hyperandrogenism. Hence, hyperinsulinemia seems to be relevant in the regulation of HR, in which it causes excessive production of androgens by theca cells and gonadotropins, causing elevated LH secretion in women with PCOS. Increased insulin levels may also cause dysregulated inhibition of GnRH by progesterone. In vitro, insulin may also cause increased adrenal steroidogenesis and enhanced secretory responses to acetylcholine [14]. Hence, both IR and hyperandrogenemia in PCOS can create a cycle in which they stimulate each other to cause deleterious effects.

To summarize, IR in PCOS can be caused by serine phosphorylation of the cytochrome p450 17ɑ-hydroxylase enzyme involved in androgen synthesis, TNF-ɑ inhibition of tyrosine kinase function of the insulin receptor, a mutation in MTNR1B, insufficient vitamin D, and a decrease in NO and an increase in endothelin 1 resulting in vasoconstriction through TGFβ. Regarding the effects of IR on PCOS, there is controversy regarding whether IR is related or unrelated to obesity and body mass indexes. Studies showed that glucose clearance, mediated by insulin, is decreased in women with PCOS, and hepatic IR is seen only in obese women with PCOS. PCOS patients have increased plasma insulin, triglycerides, and total cholesterol but decreased insulin responsiveness to carbohydrates, a decreased disposition index, and impaired liver functions. Furthermore, it can be said that IR and hyperandrogenemia in PCOS act together in a cyclic manner to stimulate each other and cause harmful effects. As mentioned before, IL-22 can play a therapeutic role in resolving metabolic dysfunctions in PCOS by reducing glucose levels and bringing back normal insulin responsiveness. It can also reverse glucose metabolism dysfunctions through the browning of white adipose tissue [43].

### Obesity and type 2 diabetes mellitus in PCOS

Obesity is not a fundamental characteristic of PCOS in women; however, it does exacerbate some of the symptoms, especially insulin resistance. Intra-abdominal fat is sensitive to lipolysis and allows for the release of free fatty acids and the synthesis of cytokines which are also involved in insulin resistance. Obese women with PCOS tend to be more abdominally obese with increased visceral fat. When obese women with PCOS are compared to non-obese women with PCOS, there are irregularities in the menstrual cycle, increased bleeding of the uterus, increased preponderance of infertility, increased hirsutism, and acne, yet decreased levels of SHBG in obese women with PCOS [6]. However, decreased SHBG levels increase the bioavailability of testosterone [6], which consequently increases hyperandrogenemia. Furthermore, obese PCOS women are at an increased risk of developing diabetes. T2DM in PCOS can be caused by insulin resistance and defective beta cell function. The onset of hyperinsulinemia and the accumulation of abdominal fat in diabetic cases in women with PCOS can also intensify hyperandrogenemia [14]. Additionally, it seems that chronic anovulation, which can cause an increase in androgen levels, is associated with lower insulin sensitivity, T2DM, and dyslipidemia. However, hyperandrogenism with polycystic ovaries or ovulation with polycystic ovaries and no hyperandrogenism in women seem to have no correlation to insulin resistance or T2DM. Epidemiologic studies have shown that the occurrence of diabetes in women with PCOS has an odds ratio of 2.0 [78]. Additionally, the cytokine IL-6 seems to have an association with inflammation, obesity by increasing the activation of the AKT pathway, and IR through excess IL-6 synthesis [40, 41].

**Fig. 10:**
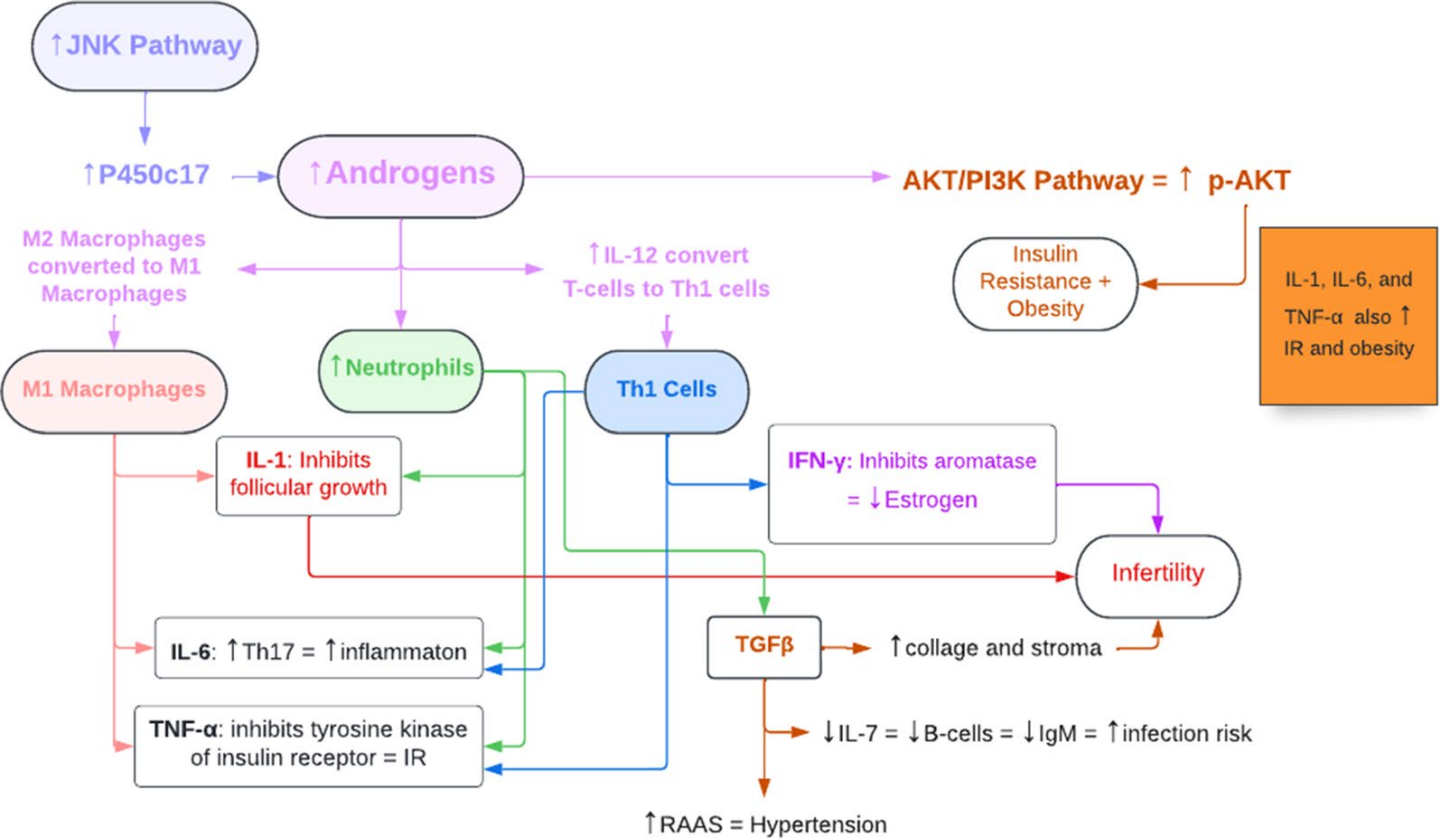
The interconnections between immune cells, signaling pathways, and cytokines. *RAAS* renin–angiotensin–aldosterone system, *p-AKT* phosphorylated AKT, *TNF-α* tumor necrosis factor-α, *IL* interleukin, *Th1 cells* T helper 1 cells, *Th17 cells* T helper 17 cells, *IFN-*γ *interferon y, TGFβ* transforming growth factor β, *IR* insulin resistance, *IgM* immunoglobulin M

A study conducted by Barcellos et al. used an oral glucose tolerance test on 85 patients who were around 25 years old with a body mass index of 28.5 ± 6.6 kg/m^2^. The researchers classified the severity of glucose tolerance based on fasting plasma glucose levels and plasma glucose at 120 min post-prandially [79]. It was concluded that patients with PCOS had a higher prevalence of impaired glucose metabolism which increased with body mass index in both criteria [79]. This study showed that the prevalence of obesity in PCOS patients plays a role in amplifying TD2M and abnormalities in glucose tolerance [79]. Overall, the effects of the immune system and androgens on PCOS are intertwined (Fig. 10). Cytokines, other components of the immune system, and androgens stimulate each other to exacerbate symptoms of PCOS such as HR, IR, obesity, and T2DM.

## The role of antioxidant-induced oxidative stress in the pathophysiology and management of polycystic ovary syndrome

Women with PCOS are found to have increased oxidative stress (OS) compared to non-PCOS women. Macrophages produce free radicals that lead to cell damage, OS, and chronic inflammation. OS is defined as an imbalance in the oxidant-to-antioxidant ratio, where the damage caused by higher oxidant levels overrides the protective measures of antioxidants in the body. Various genetic and environmental factors cause OS in PCOS. The main mechanism involves increased reactive oxygen species (ROS) production, leading to mitochondrial damage. This ROS increase may be attributed to a decreased amount of mt-DNA and mitochondrial gene mutations. A single point mutation in mitochondrial transfer RNA was identified, which contributes to metabolic dysfunction in PCOS women, and a single nucleotide polymorphism in the D loop of mt-DNA. Skov et al. reported that the electron transport from ubiquitin to Cytochrome C in mitochondria is the most dysfunctional cellular component in the skeletal muscle of PCOS women [18]. These mitochondrial alterations may result in apoptosis and decreased reproductive function [80]. Diet can also stimulate OS, where excess glucose absorption causes ROS formation and an inflammatory response. Insulin allows for ATP synthesis through oxidative phosphorylation in the mitochondria, and the mitochondria produce ROS to increase insulin sensitivity. This results in the abnormal signaling of the JNK pathway and MAPK cascade, causing increased phosphorylation of the insulin receptor. It also causes the release of Nfk-B, TNF-α, and IL-6, all of which contribute to insulin resistance in PCOS. OS can also cause obesity through adipocyte proliferation and increasing the size of mature adipocytes. Some studies indicate that ROS can stimulate neurons to induce hunger. In addition, a recent study by Abudawood et al. reported that elevated levels of heavy metals in the body cause OS in PCOS women. Increased concentrations of heavy metals influence the production of ROS, and these heavy metals act as endocrine disruptor chemicals that can lower antioxidant status by inducing OS. Women with PCOS were found to have markedly increased amounts of heavy metals such as arsenic, cadmium, mercury, and lead in serum compared to the control group. The antioxidant status was then measured by using antioxidant biomarkers, serum glutathione, and serum superoxide dismutase, both of which were decreased in PCOS women. This indicates that the increased heavy metal concentration in PCOS women contributes to higher OS and lower antioxidant levels, thus reducing the capacity to detoxify ROS from the body [81].

The increased OS caused by various genetic and environmental factors can exacerbate the symptoms of PCOS. As noted, the increased OS can lead to insulin resistance, obesity, and decreased antioxidant status. Elevated ROS can also affect oocyte maturation. Under normal conditions, the process of the dominant oocyte completing meiosis I is associated with a rise in ROS levels and inhibition of antioxidant synthesis. Meiosis II then requires antioxidant protection during division [18]. Increased ROS concentrations cause apoptosis of granulosa cells, limiting oocyte development and maturation [82]. ROS production also leads to increased secretion of intracellular calcium from the endoplasmic reticulum, which interferes with ATP synthesis, ultimately leading to cell necrosis. This dysregulation of calcium release causes follicular arrest, contributing to menstrual and reproductive abnormalities [83]. Direct oxidation of amino acid side chains leads to the formation of carbonyl products that can disrupt protein function. Plasma advanced protein oxidation products (AOPPs) are newly identified markers of oxidant-mediated cellular damage, which appear elevated in PCOS women [83]. Additionally, OS and inflammation are closely intertwined in PCOS. Several studies have found that CRP and white blood cell (WBC) count increase in PCOS women. Xanthine Oxidase (XO) also seems to be increased in PCOS, which is responsible for producing superoxide anion radicals [18]. This increase in inflammatory markers positively correlates with OS in PCOS women. Furthermore, the inflammatory cytokines IL-6, IL-8, and TNF-a are all increased, whereas the anti-inflammatory cytokine IL-10 is decreased in women with PCOS [18]. Hence, OS seems to be pivotal in the pathogenesis and exacerbation of PCOS symptoms and its associated inflammation.

OS overrides the ability of antioxidants to perform their protective functions in PCOS women. For this reason, several therapies have been identified to target this depletion of antioxidants and to ameliorate some of the complications associated with PCOS. Jamilian et al. suggested 50,000 IU of vitamin D for two weeks and 2000 mg per day of omega-3 fatty acid supplementation in PCOS women for 12 weeks. The researchers noticed a decrease in serum testosterone, CRP, malondialdehyde (MDA), and IL-1. They noted improved mental health, increased antioxidant status, and upregulated VEGF [84]. Selenium supplementation is also suggested as it has antioxidant properties and can reduce ROS formation. Combining selenium and probiotics for 12 weeks decreased MDA, CRP, testosterone, and glutathione, highlighting their antioxidant and anti-inflammatory properties [18]. Other antioxidants that have the ability to inhibit OS and ROS production include alpha lipoic acid, vitamin C, vitamin E, Coenzyme Q, N-acetylcysteine, resveratrol, melatonin, and carnitine. Using these antioxidants in combination with traditional medications that induce ovulation may improve the symptoms of PCOS and reduce both OS and inflammation [85].

Some drugs can reduce OS. Metformin has been recently reported to increase oxygen consumption by the mitochondria, resulting in elevated glutathione and decreased ROS levels [86]. Sathyapalan et al. treated PCOS patients with 20 mg of atorvastatin daily for three months and noted a decrease in MDA [87]. In addition, Eftekhari et al. brought attention to the antioxidant and anti-inflammatory properties of the Mentha species, indicating that various extracts and essential oils of the Mentha spp. scavenge free radicals such as hydroxyl, nitric oxide, hydrogen peroxide, and superoxide radicals. The free radical degrading properties of Mentha spp are due to their ability to chelate metals and to be donors of hydrogen or electrons. The anti-inflammatory properties come from the ability of the Mentha spp. to inhibit 5-lipoxygenase, which will then inhibit the production of nitric oxide and prostaglandin E2. The Mentha spp. also decreases the production of IL-1, IL-6, and COX-2 [88]. A study by Atabaadi et al. treated PCOS-induced rats with 150 mg/kg or 300 mg/kg of spearmint oil; the spearmint oil-treated rats had decreased body weight, testosterone, and ovarian cysts but presented with increased Graafian follicles [89]. This suggests that the Mentha spp could be a potential treatment for women with PCOS. Lastly, some research suggests that antioxidant therapy is less effective due to its decreased bioavailability and low absorption profiles. Metallic nanoparticles, commonly referred to as nano-antioxidants, have become a novel treatment option for OS-related disorders. Nano-antioxidants are artificially synthesized materials, such as “magnetic, silver and gold” [90] metallic nanoparticles, that have increased bioavailability, surface area and catalytic reactivity compared to traditional antioxidants and should be considered for the treatment of PCOS complications. However, a common adverse effect is nano-antioxidant toxicity which can result in the overproduction of ROS by inhibiting the electron transport chain in the mitochondria. Toxicity can also cause overexpression of inflammatory cytokines and cells, further increasing ROS synthesis. For this reason, “maximal effective doses” [90] of nano-antioxidants should be determined to prevent adverse effects.

In summary, OS is a pivotal contributor to the pathophysiology of PCOS. OS, indicative of the complex nature of the immune system in PCOS women, can induce inflammation. PCOS women have diminished antioxidant levels which have been attributed to the exacerbation of insulin resistance, obesity, hyperandrogenism, reproductive dysfunction, and chronic inflammation. This dysregulated oxidant-antioxidant ratio highlights the opportunity for novel treatment options in women with PCOS. Management could focus on reducing ROS production with antioxidant supplementation, drugs such as metformin, and extracts of the Mentha spp. However, it is important to note that further robust clinical studies that are sufficiently powered using standardized interventions are needed.

## Future perspectives

**Fig. 11:**
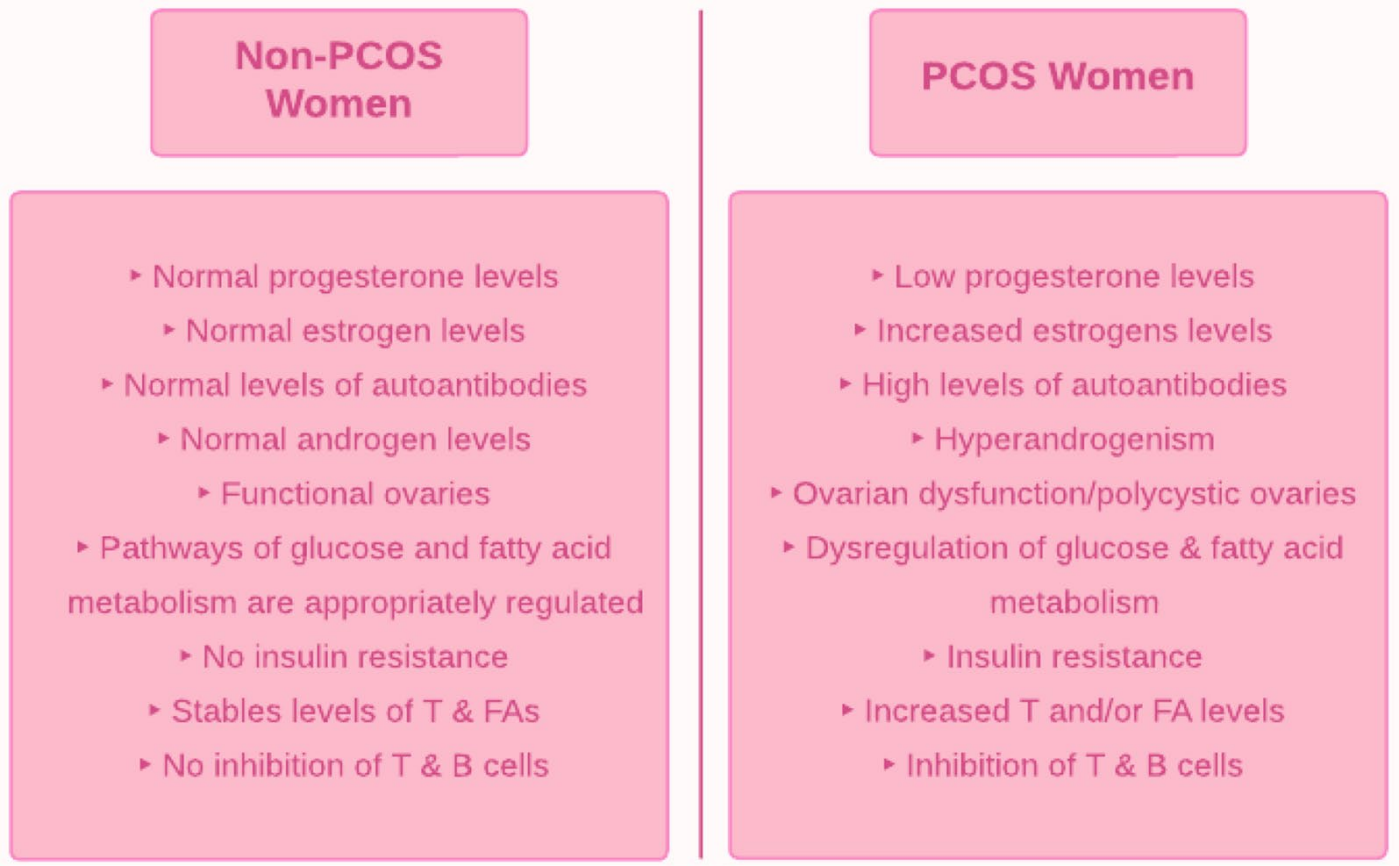
An overview of the differences between PCOS women and non-PCOS women. *T* testosterone, *FA* fatty acids

Recent studies have suggested that PCOS could possibly be an autoimmune disorder. For example, a study reported a high presence of antiovarian antibodies in PCOS, which suggests a relationship between immune cells and PCOS (Fig. 11) [91]. Further research into this relationship with the immune system could result in a greater understanding of PCOS, as well as new possible treatments. However, significantly more research is needed.

Several autoantibodies have been reported in PCOS, such as anti-nuclear (ANA), anti-thyroid, and anti-ovarian autoantibodies. These autoantibodies are present due to low progesterone levels in PCOS, which causes over-activation of the immune system. This then leads to increased levels of estrogen, resulting in numerous autoantibodies [92]. It is suggested that future research is required on the fluctuating levels of autoantibodies in PCOS that could possibly lead to future treatments for PCOS and that may be more effective than what is currently used today [92]. One analysis reported that a majority of people with an autoimmune disorder are women, and this may be related to estrogen levels because the onset of autoimmune disorders occurs earlier in age for women compared to men [24]. Further investigation of the reasons why autoimmune disorders are more prominent in women could possibly give insight into PCOS as an autoimmune disorder.

The insulin-sensitive drugs metformin and rosiglitazone decreased androgen levels in PCOS women [6]. Furthermore, a study showed that the administration of IL-22 in mice revealed that IL-22 had several effects. These effects included improved ovarian function, decreased androgen levels, and reversed metabolic dysfunction [43]. With this information, researchers could compare these substances to try to determine if one has a greater therapeutic effect than the other. IL-22, in particular, has various effects, and more experiments could be conducted to possibly create a treatment for PCOS or at least open a new gateway of exploration for novel drugs.

In a recent study, obese PCOS patients were treated with l-arginine, *N*-acetyl-cysteine, l-carnitine, and acetyl-l-carnitine. This resulted in decreased plasma insulin, triglyceride, and total cholesterol levels [77]. It can be concluded that treatment with l-arginine, *N*-acetyl-cysteine, l-carnitine, and acetyl-l-carnitine can therefore manage hyperinsulinemia. Further studies with these substances could lead to new and improved management plans for hyperinsulinemia in PCOS patients.

Regarding OS present in PCOS, diet is a contributing factor. Excessive glucose results in ROS formation, thus increasing insulin sensitivity and causing obesity [81]. Furthermore, vitamin D has been shown to protect against OS by controlling inflammation and promoting mitochondrial function. Researchers have tested that vitamin D given to PCOS patients results in a decrease in serum testosterone, CRP, MDA, and IL-1 [84]. In addition, selenium supplementation and probiotics have been shown to decrease MDA, CRP, testosterone, and glutathione, leading to antioxidant and anti-inflammatory properties [18]. On the other hand, potential medications have been found to reduce OS, such as Metformin, which has been shown to decrease ROS levels [86]. From these tests, it can be deduced that diet control and supplementation contribute significantly to PCOS patients. PCOS patients can be provided a diet plan that ensures they regularly consume foods containing vitamin D and selenium and control their glucose intake. Future research could examine how significant a changed diet would be in PCOS patients. In addition, PCOS patients could take OS-reducing medication alongside diet changes to further reduce ROS levels.

The possibility of PCOS being an autoimmune disorder is still poorly delineated. Despite there being several connections made between PCOS and the immune system, there are countless unanswered questions, and further research is required. This is a vital area of exploration, as PCOS is extremely common, and a better understanding of the underlying mechanisms and more efficient treatments would be extremely beneficial.

## Conclusion

The inter-relationship of androgens and immunology in PCOS is multifaceted. For this very reason, the Rotterdam criteria have been developed over the years to diagnose PCOS. PCOS and, consequently, HR have unfavorable impacts on the lives of many women. Hypersecretion of androgens results in IR, obesity, T2DM, oligomenorrhea, alopecia, acne, hirsutism, and other symptoms that can impact the quality of life. Additionally, HR and several of these symptoms seem to act in a vicious cycle in which they stimulate one another to cause deleterious effects. The pathophysiology of PCOS and HR still remains unclear due to the broad array of clinical manifestations that arise from the complex web of interactions between androgens and insulin, adipocytes, cytokines, signaling pathways, and immune cells. Despite the increasing prevalence of PCOS worldwide, many aspects of this disorder remain ambiguous, and continuing research aims to understand its complexities and characteristics. An opportunity exists to direct future research efforts toward the intricacies of immunology and androgens in PCOS. Novel treatments for PCOS can then be determined based on an understanding of immune cells and the fluctuating levels of autoantibodies in women with PCOS.

## Data Availability

No novel data were produced in the writing of this review article.
